# Enhancing the Diffusion Channels of Silica‐Alginate Capsules for Microbial Encapsulation

**DOI:** 10.1002/elsc.70002

**Published:** 2025-09-25

**Authors:** Bilyamin Abdulmumin, Ismaila Mudi, Abdulalim Ibrahim, Abdulwasiu Abdurrahman, Helen Onyeaka

**Affiliations:** ^1^ Department of Chemical Engineering Faculty of Engineering Ahmadu Bello University Zaria Nigeria; ^2^ School of Chemical Engineering University of Birmingham Edgbaston Birmingham UK; ^3^ Chemical Engineering Department Federal University Wukari Wukari Nigeria; ^4^ IMT Mines Albi UMR CNRS 5302 Centre RAPSODEE Universite de Tolouse Albi France; ^5^ Department of Chemical Engineering Faculty of Engineering University of Abuja Abuja Nigeria

**Keywords:** Ca‐alginate capsule, diffusion coefficient, fermentation, microbial encapsulation, porous silica coating

## Abstract

Silica‐alginate capsule (G‐0) has recently been used in fermentation processes to encapsulate microbial cells for several benefits, including facilitating continuous flow processes and simplifying cell recovery and reuse. However, these conventional silica‐coated alginate capsules suffer from poor diffusion channels, which are critical for efficiently transporting substrates and products. This study aimed to develop a novel method for producing silica‐coated alginate capsules with improved diffusion channels (G‐3). The Ca‐alginate capsule was fabricated via a simple dripping method, where a solution of calcium chloride (CaCl_2_) and carboxymethylcellulose (CMC) was dripped into an alginate solution. For the traditional silica coating (G‐0), the alginate capsule was mixed with a silica source (hydrolyzed 3‐aminopropyl triethoxysilane) under specific conditions. In the modified method, glucose was introduced as a pore‐forming agent (PFA), with varying amounts (0.75, 1.5, and 3 g) resulting in capsules labeled G‐0.75, G‐1.5, and G‐3, respectively. The diffusion coefficient for G‐3 was found to be the highest, for example, at 313.15 K, it was calculated as $\;\left(7.77\pm 0.57\right)\times 10^{-3}\;mm^{2}/\min$ compared to $\left(3.04\pm 0.09\right)\times 10^{-3}mm^{2}/\min$ for G‐0. This finding highlights the effectiveness of PFA in enhancing membrane porosity and diffusivity, which is promising for microbial cell immobilization where mass transfer is a significant concern.

**Abbreviations**
ANOVA: analysis of variance
APTES: 3‐aminopropyl triethoxysilane
Avg: average
CaCl_2_
: calcium chloride
CFU: colony‐forming units
CMC: carboxymethylcellulose
DNSA: 3,5‐dintitrosalicylic acid
HSD: honestly significant difference
PFA: pore‐forming agent
SDA: sabouraud dextrose agar
SE: standard error
Sim: simulated
TEOS: tetraethyl orthosilicate

## 1 Introduction

Microbial cells are encapsulated for various benefits. Some of these benefits include facilitating easier recovery and reuse of cells, improving cell tolerance, and allowing for continuous flow fermentation [1, 2, 3, 4, 5].

**Summary**

- These capsules are employed for bioethanol production where mass transfer of substrate and product is critical.
- Beyond biofuels, this method could be extended to wastewater treatment for the degradation of toxic chemicals, pharmaceuticals for controlled drug delivery, agriculture for fertilizer control release, food and beverages for aroma and oil control release or biomass protection, and biocatalysis for enzyme immobilization.
- This research provides a foundation for developing next‐generation encapsulation technologies that address a long‐standing challenge: combine stability with high diffusivity.

Alginate biopolymer is widely used for microbial cell encapsulation [6, 7, 8]. Its popularity stems from its affordability and availability and its simplicity in forming an ionic gel when interacting with divalent cations such as calcium ions. Ca‐alginate capsules are formed by dripping a mixture of Ca^2^⁺ ions and encapsulating cells into an alginate polymer solution. The calcium ions rapidly diffuse into the alginate, forming electrostatic bonds that result in a semipermeable membrane encapsulating the cells [9].

The advantages of Ca‐alginate capsule reusability were exemplified by Talebnia et al. [10]. The authors conducted 10 sequential batch fermentations using the same yeast Ca‐alginate capsule, yielding consistent bioethanol productivity and yield across all batches. The authors further compared free and encapsulated cells in highly toxic hydrolysate for bioethanol production; the study revealed that encapsulated cells converted hydrolysate in less than 10 h, while free cells could not convert the same hydrolysate in 24 h. This is an indication of improved tolerance of encapsulated cells to fermentation conditions. Moreover, cell encapsulation improves stress tolerance against elevated temperatures, allowing performance up to 45°C, compared to the mesophilic temperature of 37°C for free cells [11]. So, simultaneous saccharification and bioethanol production, which are challenging due to differing operating temperatures between saccharification and fermentation enzymes, become feasible. Najafpour et al. [12] demonstrated that encapsulated yeast could convert high glucose concentrations up to 150 g/L to bioethanol, whereas free cells could not ferment glucose concentrations of about 100 g/L; furthermore, since the process was carried out as a continuous process, it indicated the possibility of a continuous fermentation process.

However, despite the aforementioned benefits, Ca‐alginate capsule application is limited due to instability for long or repeated deployment. The interaction between calcium ions and alginate is primarily electrostatic and relatively weak, making the structure prone to distortion in the presence of chelating agents, such as phosphates or citrate; these agents can compete with alginate for binding to calcium ions which leads to the dissolution of the crosslinked network [13]. Furthermore, in applications such as fermentation, mechanical forces like agitation and shear stress can disrupt the Ca‐alginate binding, causing deformation or breaking of the capsules. To address this, silica coating has been extensively researched [9, 14, 15]. Sol–gel technology enables the synthesis of silica on alginate capsules under mild operating conditions [14]. Various silica precursors are used, including silicon alkoxide, modified silicon alkoxide, and non‐silica silicon alkoxide [16, 17].

Dragomirescu et al. [18] employed tetramethoxysilane (TMOS) and tetraethoxysilane (TEOS) as silica sources for the enzyme release system. Instead of alkoxide, Sakai et al. [19] employed 3‐aminopropyl triethoxysilane (APTES, modified alkoxide) as a silica source for drug delivery systems. Rehbein et al. [20] showed that using a combination of APTES and TEOS as a silica precursor reduces the time of silica gelation. Wen et al. [21] as well as Miloudi and Zerrouki [22] employed non‐alkoxide (sodium silicate) as a silica source for intestinal‐targeted drug release and phase change materials (PCMs) encapsulation, respectively. Colloidal silica was another non‐alkoxide silica source employed by Shi et al. [23] for the efficient removal of phenol from wastewater.

Each silica source has particular pros and cons depending on application, from an environmental and economic perspective, modified silicon alkoxide as a silica source, such as 3‐amino tetraethoxysilane (APTES), is preferred [24]. In addition, the APTES adsorption process on the surface of alginate does not require an organic solvent for dissolution, nor does it necessitate prior modification of the capsule surface [17].

APTES solution bears a positive charge at pH levels below 9.6, the p*K*_a_ of the amino‐functional group in APTES. Conversely, the alginate capsule bears a negative charge at pH levels above 3.56, the dissociation constants (p*K*_a_) of alginate monomers G and M [25]. Therefore, electrostatic crosslinking between the alginate and APTES occurs in a medium with a pH range between 9.6 and 3.56.

Two main methods to achieve silica coating with APTES are layer‐by‐layer and coacervation methods. In the layer‐by‐layer approach, the alginate capsule is first fabricated, followed by silica coating as an additional layer on the microcapsule surface [14]. In the coacervation (impregnation) method, capsule fabrication and silica impregnation occur simultaneously [17]. Although the layer‐by‐layer method synthesizes silica as an additional layer, the impregnation method incorporates the silica within the capsule. From a microorganism‐friendly operational perspective, layer‐by‐layer silica coating is preferred since it reduces direct contact with inorganic coating materials.

Although coating Ca‐alginate capsules is reported to improve their stability, it has a limitation: it reduces the capsule's porosity [26]. This is a challenge in fermentation processes where high diffusion of substrate and product is crucial [27]. Therefore, a stable capsule with better porous membranes is needed.

Hence, this study reports a novel method for coating Ca‐alginate capsules with higher porous silica for applications in microbial encapsulation. APTES and glucose were used as the silica precursor and pore‐forming agent (PFA), respectively. The morphology, as well as stability of the capsules developed, was studied, and MATLAB code was written to fit the experiment data, determine the diffusion coefficients, and compare the experiment with the predicted result; finally, the capsules were applied for *Saccharomyces cerevisiae* encapsulation.

## 2 Materials and Methods

### 2.1 Materials

The APTES was purchased from Sigma–Aldrich (Germany). Complete APTES hydrolysis was achieved by adding an aliquot of 10 mL of APTES to 100 mL of water, and the mixture was stirred overnight to ensure complete hydrolysis. Sodium alginate, carboxymethylcellulose (CMC), calcium chloride (CaCl_2_), Tween 20, glucose, and all other chemicals used in the experiments were analytical reagent grade and were used without further purification.

### 2.2 Methods

#### 2.2.1 Calcium‐Alginate Formation and Silica Coating

According to Talebnia et al. [10], a solution of 50 mL (1.3% [w/v]) CaCl_2_ that contained CMC of 1.3% (w/v) concentration was dripped into a stirred mixture of alginate solution of 0.6% (w/v) and 0.1% (v/v) Tween 20. A syringe pump (341A, Sage Instrument, USA) was used as dripping equipment, the flow rate of 0.51 mL/min was maintained, and 7 cm was kept as the distance between the dripping nozzle and alginate mixture. The capsule developed was allowed to cure for 10 min, then washed with distilled water and hardened in 1.3% (w/v) CaCl_2_ for another 20 min and was then collected by filtration. The obtained alginate capsule was represented as Ca‐alginate and characterized by 10× microscopy (Kenis, Model‐LB, Japan, 10×), and an iPhone 7 camera.

Before silica treatment, the pH of the hydrolyzed APTES solution was adjusted to a value of 5 using 0.2 M HCl. For the traditional silica coating, 15 capsules were mixed with 30 mL (3% w/w) of the hydrolyzed APTES in a 250 mL conical flask at 30°C and constant agitation (130 rpm) on a magnetic hotplate stirrer (SB‐162‐3, Stuart, UK) for 90 min. The traditional capsules were collected by filtration and stored in a 250 mL beaker containing distilled water at room temperature. The capsules were referred to as G‐0 and characterized by 10× microscopies (Kenis, Model‐LB, Japan), iPhone 7, stability, and diffusion test. The modified silica coating was carried out similarly to the traditional silica procedure, except that a PFA (glucose) was included in this route. The glucose concentration was varied (0.75, 1.5, and 3 g). After coating, the glucose was removed through water extraction by immersing the capsules in a 250 mL beaker at room temperature for 24 h. The set of capsules were designated as G‐0.75, G‐1.5, and G‐3, then characterized just as above.

#### 2.2.2 Diffusion Studies

For the diffusion tests, the different capsules prepared (G‐0, G‐0.75, G‐1.5, and G‐3) were first equilibrated with water and then immersed into 100 mL of 1.0 g/L glucose concentration. The change in glucose concentration was monitored until equilibrium was reached with the sample withdrawn at 15‐min intervals. To determine the glucose concentration that diffused into the capsule sample, the change in glucose concentration monitored was subtracted from the total glucose concentration before the diffusion took place (details data is in the Supporting Information Section A). Employing MATLAB, the codes were written to fit the experiment data into Equation (3), determine the best diffusion coefficient (D) for each sample, and finally compare the experiment data with the predicted results. Fick's second law for the case of diffusion through a particle is given by Equation (1)

(1) $$\;\frac{dCs}{dt}=D\frac{\partial^{2}Cs}{\partial r^{2}}$$

where *Cs* is the glucose concentration inside a capsule, *D* is the diffusion coefficient, *t* is the time, and *r* is the radial distance from the center of the capsule. For a spherical geometry, Equation (1) becomes Equation (2)

(2) $$\frac{dCs}{dt}=D\left(\frac{1}{r^{2}}\cdot \frac{d}{dr}\left(r^{2}\cdot \frac{dCs}{dr}\right)\right)$$

Equation (2) is a partial differential equation that can be discretized using the central difference approximation method into Equation (3)

(3) $$\;\frac{dCs}{dt}=D\left(\frac{Cs_{i+1}-Cs_{i-1}}{r_{i}\Delta r}+\frac{Cs_{i+1}+2Cs_{i}-Cs_{i-1}}{\Delta r^{2}}\right)$$

The detailed MATLAB code written to fit experiment data, find the best *D*, predict results, and compare experiments with predicted results for each dataset is given as Supporting Information (Section A). Since the experiment was carried out in duplicate, the Supporting Information gives the code that finds the average (Avg) and standard error (SE) as well as the *R* ^2^ for the two experiments (Supporting Information Section B).

Furthermore, the diffusion coefficient dependency on temperature was analyzed according to the Arrhenius Equation (4)

(4) $$D=D_{0}\;\mathrm{EXP}\left(\frac{E_{a}}{RT}\right)$$

where *D*_0_ is the pre‐exponential factor, *E*_a_ is the diffusion's activation energy (kJ/mol), *R* (8.314 kJ/mol K) is the gas constant, and *T* is the Kelvin temperature. Hence *D* of each sample was calculated at a temperature range of 20, 35, and 40°C, finally, the activation energy for each sample was calculated using the equation. Supporting Information Section C gives the detailed data and plots for the set of experiments.

#### 2.2.3 Stability Studies

The mechanical stability of the capsules against shear forces was measured by adding 20 capsules to a 400 mL conical flask. The capsules were mixed vigorously at 400 rpm at room temperature, and the number of broken capsules was monitored for 5 h. Stability efficiency (%) was determined by dividing the number of capsules broken by the original number of capsules and multiplying by100.

#### 2.2.4 Microbial Cell Encapsulation and Viability Study

About 50 mL of *S. cerevisiae* cells were encapsulated according to the procedure mentioned, and the number of encapsulated cells was increased by aerobic fermentation. Fifteen capsules (about 12.5 mL) of each sample (G‐0, G‐0.75, G‐1.5, and G‐3) were incubated with 100 mL glucose solution (50 g/L), containing 1% CaCl_2_ in a reactor (250 mL conical flask covered with cotton) for a fermentation period of 24 h at a temperature of 30°C and 130 rpm. Then the viable colony of the cells inside each capsule sample was determined by the spread plate method. A 10‐fold dilution of 2 mL of an aliquot of the yeast obtained from the crushed and washed capsule was made, and then 0.1 mL of the sixth dilution was spread on the surface of freshly prepared sabouraud dextrose agar (SDA) media. The discreet colonies were counted and expressed as colony‐forming units (CFU/mL). The viability of cells before all the silica treatment was determined to be 6 × 10^5^ CFU/mL).

#### 2.2.5 Analytical Method and Statistical Analysis

The glucose concentration change for different capsules was determined via spectrochemistry using the 3,5‐dintitrosalicylic acid (DNSA) method. An aliquot of DNSA reagent (0.3 mL) was mixed with an equal sample volume in a test tube. The mixture was heated for 5 min, and then a volume of 0.6 mL was measured in a cuvette to read the absorbance using a spectrophotometer (752N, Thermos Fisher, USA) at 540 nm wavelength.

All the experiment in this study was carried out in duplicate and the results were first analyzed using SE as given by Equation (5)

(5) $$\mathrm{SE}\;=\frac{\sigma }{\sqrt{n}}$$

where SE is the standard error, σ is the standard deviation, and n is the number of the samples.

One‐way and subsequent pos‐hoc (Tukey honestly significant difference [HSD]) analyses were also carried out to analyze the significance of data difference between groups, the MATLAB code for analysis of variance (ANOVA) analysis was given as Supporting Information Sections D and E for diffusion studies and stability test, respectively.

## 3 Results and Discussion

### 3.1 Silica Coating and Capsule Morphology

**FIGURE 1:**
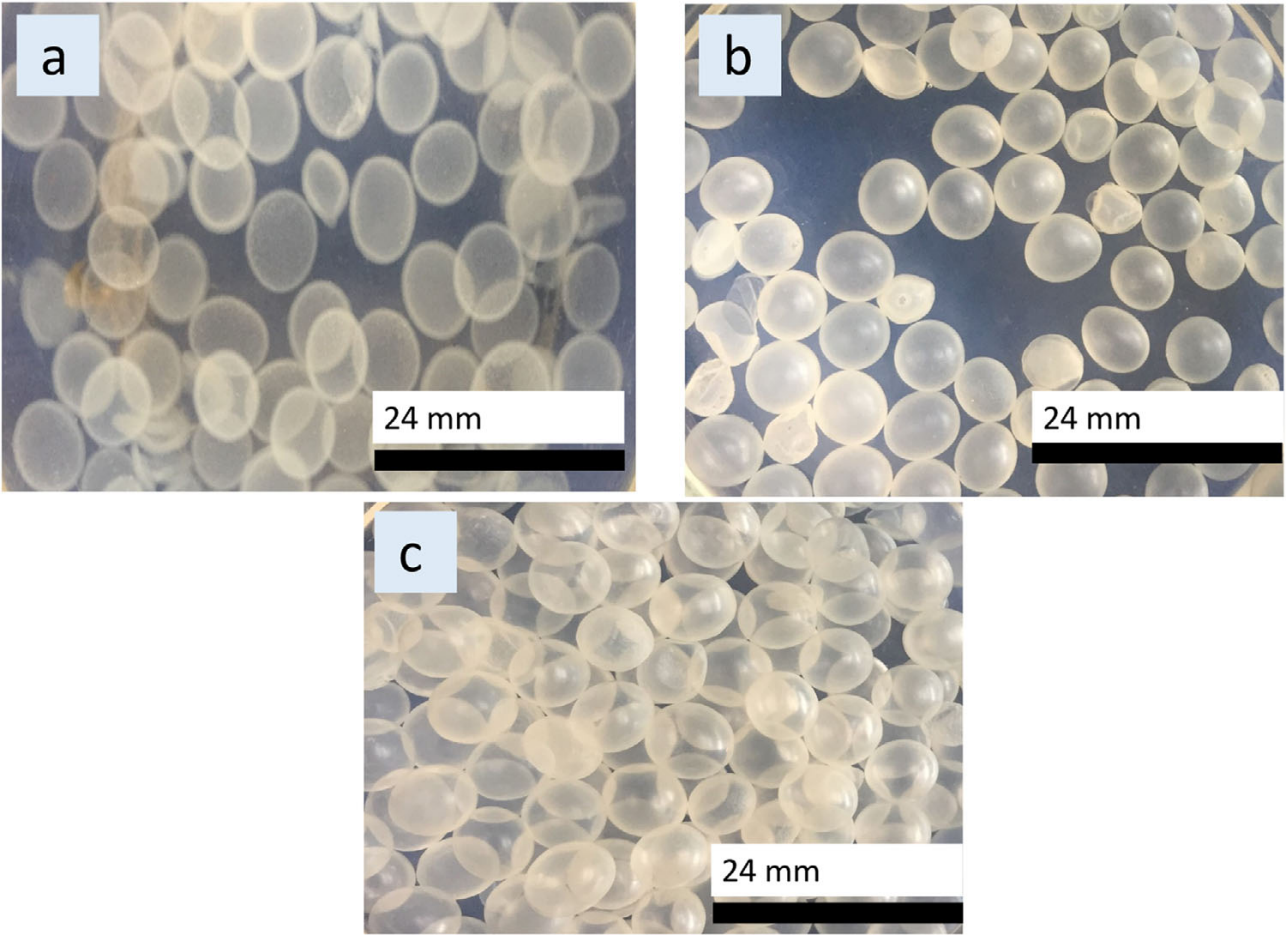
Photographs of (a) Ca‐alginate, (b) modified silica‐coated Ca‐alginate (G‐3), and (c) traditional silica‐coated Ca‐alginate (G‐0).

The Ca‐alginate was fabricated by dripping a mixture of CaCl_2_ and CMC into a solution of alginate containing Tween 20 to encapsulate the solution of CMC. When the CaCl_2_ and CMC mixture flows out of the tube opening, a droplet is formed at the tip, and the droplet grows in size until it detaches from the tip and falls inside the alginate solution. The calcium ion within the droplet diffused out the droplet to crosslink with the alginate acid to form a membrane, encapsulating the solution of CMC. A height of 7 cm between the tip of the tube and the alginate solution was maintained. Because within a distance of 7–10 cm, at 0.51 cm^3^/min, the liquid droplet can overcome the impact and drag forces of the alginate solution to form spherical gel particles [25]. After the fabrication of Ca‐alginate (Figure 1a), traditional silica coating was carried out to produce G‐0 (Figure 1c), which serves as a reference. As the Ca‐alginate was mixed with the silica source (hydrolyzed APTES), evidence of silica deposition could be physically observed, as shown in Figure 1b, c. The membrane looks opaque compared to the Ca‐alginate membrane, which is transparent. The traditional silica‐coated Ca‐alginate, G‐0, can be seen to be the opaquest, indicating the highest silica concentration. Using a Vernier caliper, the Avg diameters of the three capsules, Ca‐alginate, G‐3, and G‐0, were calculated to be 6.02, 5.90, and 5.91 mm, respectively. Like the finding in another study [24], this indicates that silica coating shrinks the capsule membrane.

**FIGURE 2:**
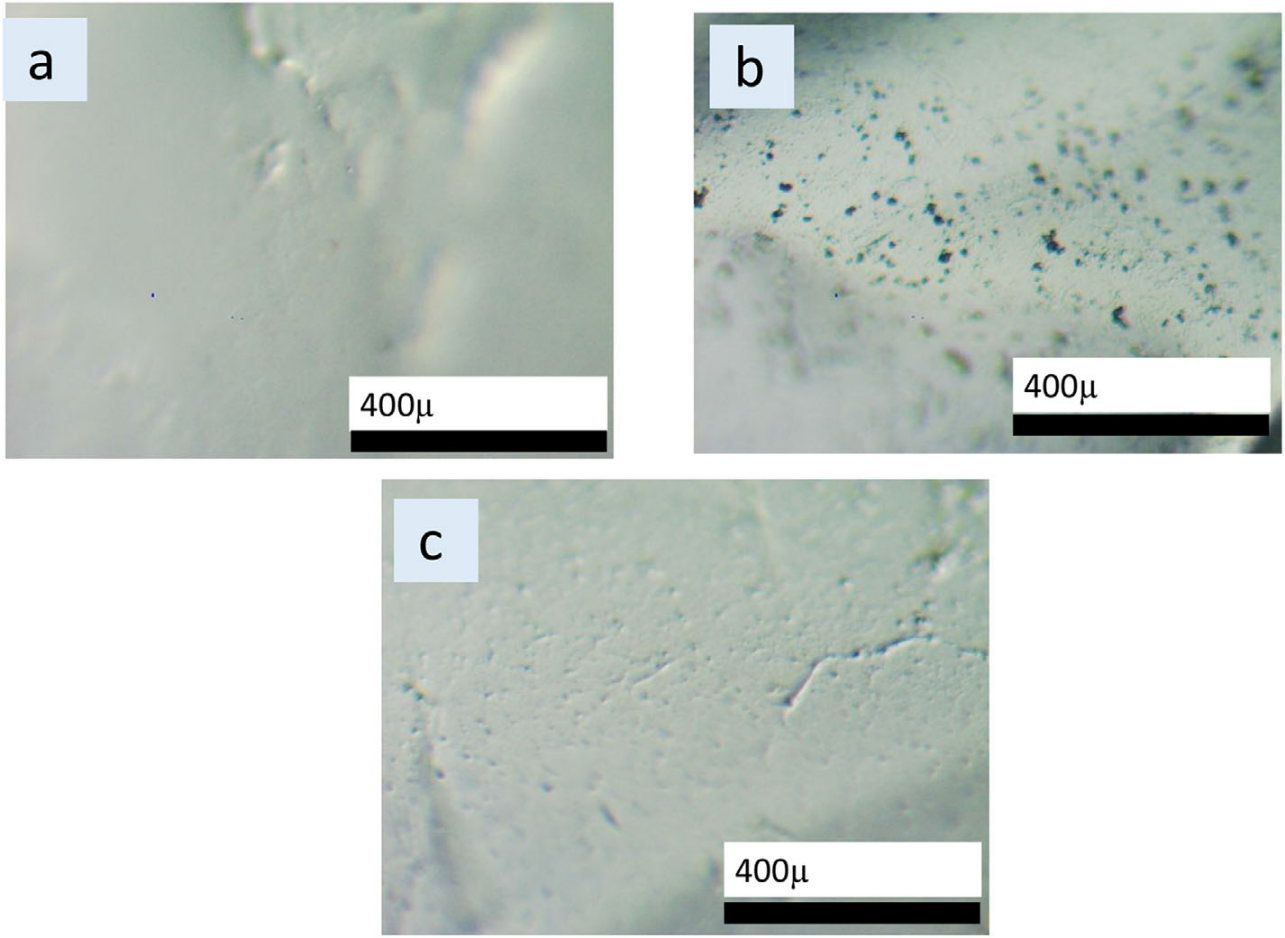
Microscopic image of (a) Ca‐alginate, (b) traditional silica‐coated Ca‐alginate (G‐0), and (c) modified silica‐coated Ca‐alginate (G‐3).

However, to better observe the Capsules’ surface morphology, the microscopic images are captured and presented in Figure 2. The uncoated Ca‐alginate shows the relatively smoothest surface, which indicates a lack of silica trace on the surface. In Figure 2b, the unmodified silica‐coated capsule surface shows the highest coarse surface, which indicates the most silica agglomeration. Although the silica agglomeration is observed on all the capsules (G‐0, G‐0.75, G‐1.5, and G‐3, refer to Supporting Information Section F) it is more noticeable on G‐0. The observed difference may be due to the influence of glucose during the silica coating process. In the case of G‐0.75, G‐1.5, and G‐3 capsules, the presence of glucose during the silica coating process inhibits silica particle agglomeration compared to G‐0 where no PFA was involved [28, 29]. Glucose likely interacts with silica precursors, through hydrogen bonding, leading to a more uniform deposition. For G‐0 capsules, the absence of glucose allows the silica precursors to agglomerate more freely.

The deposition of silica on the surface of alginate can be attributed to the crosslinking between deprotonated alginate and protonated APTES. At pH values above the dissociation constants (p*K*_a_) of alginate monomers G (3.56) and M (3.38) [25], alginate carries a negative charge. Conversely, at pH values below the p*K*_a_ of the amino‐functional group in hydrolyzed APTES (9.6), APTES becomes positively charged. This difference in charge facilitates the electrostatic interactions between alginate and APTES. Following the adsorption, the condensation of silanol groups occurs according to Equation (6), forming a porous silica gel on the Ca‐alginate surface. Therefore, silica coating using APTES as a silica source can occur at any pH between the p*K*_a_ values of alginate (3.56) and APTES (9.6), except at the zwitterion formation pH of APTES, which is 6.7. The zwitterionic nature of APTES at pH 6.7 makes it highly unstable which leads to the formation of the highest number of oligomers, this in turn reduces the amount of hydroxyl groups available for interactions [30]. In this study, a pH of 5 was chosen because APTES is reported to be more stable in an acidic environment [31]

(6)

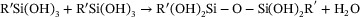




In addition, in Figure 2c, the modified silica‐coated capsule surface revealed a less coarse surface compared to Figure 2b. This indicates that, as mentioned, the presence of a PFA reduces the agglomeration of silica on the surface of the gel.

### 3.2 Diffusions Studies

Silica coating on the Ca‐alginate has been reported to reduce the capsule porosity, for instance, Choudhari et al. [26] found the diffusivity of water in a Ca‐alginate to be 0.6 mm^2^/min but when coated with silica the diffusivity reduced to 0.38 mm^2^/min, the reduction was even much more observed with isopropanol molecules as its diffusivity for Ca‐alginate and Ca‐alginate/silica were found to be 5.4 and 0.17 × 10^−3^ mm^2^/min, respectively. This informed the aim of why this study was undertaken to improve diffusional channels of Ca‐alginate‐silica capsules.

**FIGURE 3:**
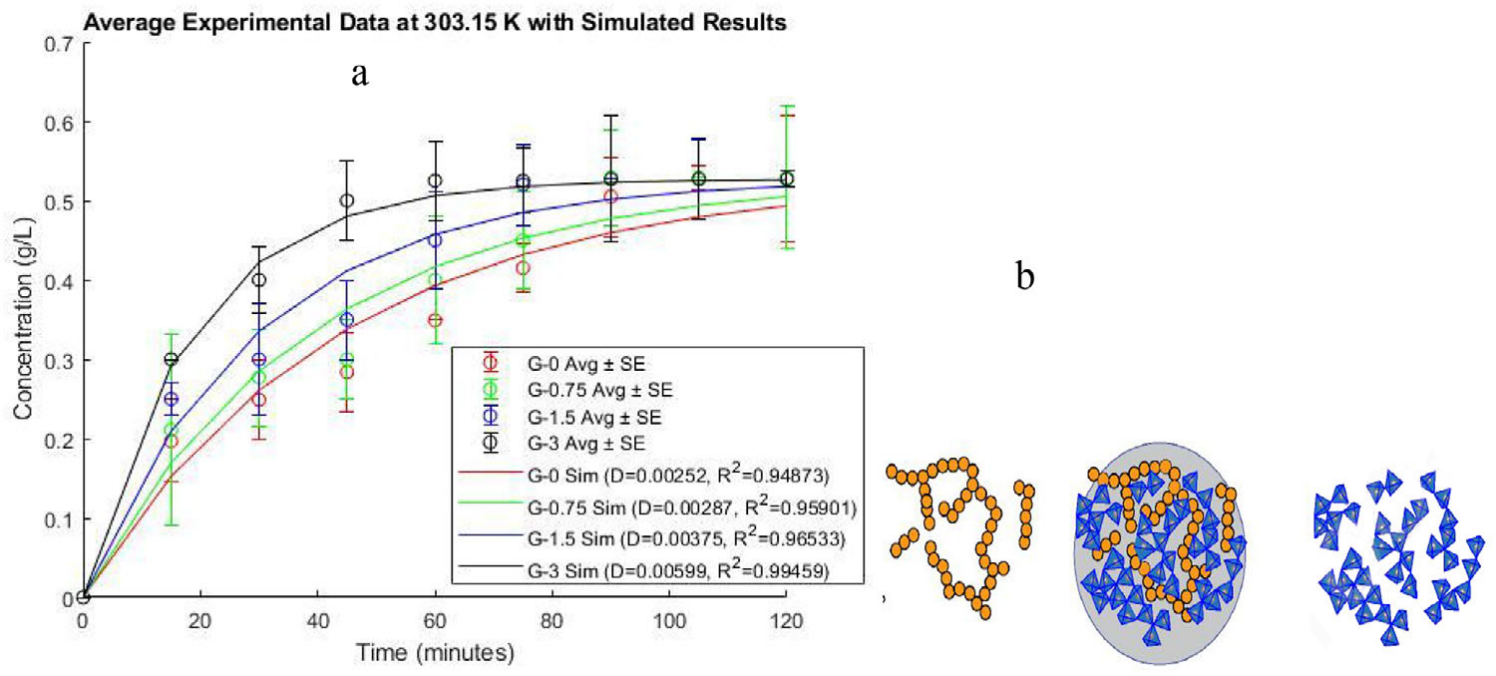
(a) Comparison between experiment and predicted data for an increase in glucose concentration with time inside different capsule samples at 30°C and 150 rpm (b) scheme for enhancing the silica‐coated Ca‐alginate surface (blue) using glucose (orange) as pore‐forming agent.

So, to determine the effect of PFA on enhancing the diffusion, the diffusivity of the four capsule samples, G‐0, G‐0.75, G‐1.5, and G‐3, was assessed from the equilibration test, where time to reach equilibrium for glucose mass transfer across the capsules was monitored. These data were then modeled and simulated (Sim) as given in Figure 3a. From the experimental data, it can be observed that the rate of glucose concentration inside the capsule increases with time and with the amount of PFA used. For instance, the capsule (G‐3), whose membrane was modified with the highest PFA concentration, showed the highest diffusivity and reached equilibrium for about 45 min. This indicates improved diffusion channels to the glucose molecules compared to the sample without PFA (G‐0), in which glucose mass transfer barely reached equilibrium for 120 min.

MATLAB function lsqcurvefit was employed to fit the experiment data, using Fick's second law equation (detailed experiment modeling and simulation codes are given as Supporting Information Section B). From the simulation result, the molecular diffusion coefficient for G‐0 and G‐3 was determined to be $\left(2.525\;\pm 1.02\right)\;10^{-3}mm^{2}/\min$ and $\left(5.99\;\pm 1.02\right)\times \;10^{-3}\;mm^{2}/\min$, respectively (Figure 3a), this represents a significant increase of about 2.4 times. This indicates that the modification of the Ca‐alginate‐silica capsule has been successfully achieved using glucose as a PFA.

The possible explanation for glucose acting as a PFA is that silica condensation and polycondensation occur in the presence of glucose aggregate (Figure 3b). When the glucose aggregate is extracted, it leaves a more porous silica scaffold compared to the traditional silica coating [29, 32].

**FIGURE 4:**
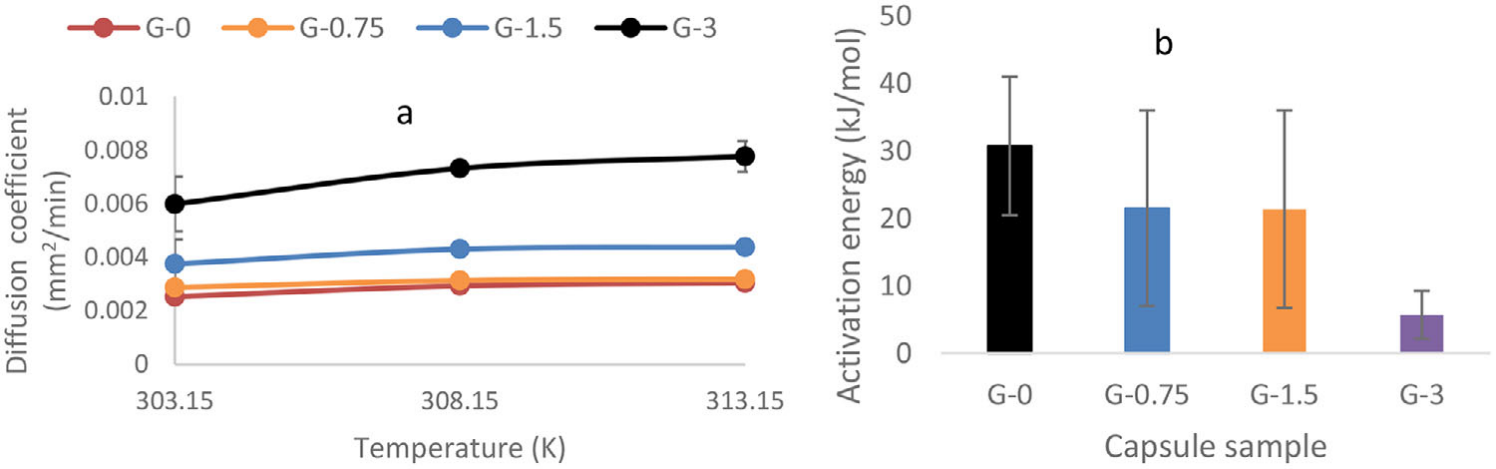
(a) Change in diffusion coefficients with temperature, (b) activation energy (*E*_a_) for the four capsule samples.

The diffusion coefficient was reported to be independent of process conditions except for temperature [33], hence this report further investigated the diffusivity dependence on temperature. The temperature varied within the range of 30, 35, and 40°C (303.15, 308.15, and 313.15 K), like in previous studies [33], the diffusion coefficient was found to increase with temperature increase (Figure 4a). Although at 303.15 K the diffusivity of the G‐0 was found to be $\;\left(2.525\;\pm 0.25\right)\times \;10^{-3}mm^{2}/\min$ but increasing temperatures to 313.15 K, the diffusivity would significantly be increased to ($3.04\;\pm 0.09)10^{-3}m$ m^2^/min. A similar trend was observed for G‐3. At 303.15 and 313.15 K, the diffusivities were ($5.99\;\pm 1.02)\;\times \;10^{-3}\;mm^{2}/\min$ and (7.77 ±0.57) × 10^−3^ mm^2^/min, respectively.

To further analyze these differences statistically, a one‐way ANOVA was conducted (Table 1). The results show that the mean difference in diffusion coefficients at 303.15 K is not statistically significant (*p* value was 0.0762). However, at higher temperatures, such as 308.15 and 313.15 K, the *p* values were 0.000 and 0.0010, respectively, indicating significant differences. Post‐hoc analysis (Figures D1–D3, Supporting Information Section D) illustrates the extent of these mean differences. Although an impressive difference between G‐0 and G‐3 was achieved at 303.15 K, statistics show that there is no significant difference, except at higher temperatures. Hence comparison of the diffusion coefficient between G‐0 and G‐3 at 308.15 or 313.15 K is more appropriate.

**TABLE 1:** Analysis of variance, *p* value, and mean difference of diffusivities coefficient.

Temperature (K)	*p*‐value	Mean difference
303.15	0.0762	Not significant (*p* > 0.05)
308.15	0.0000	Significant (*p* < 0.05)
313.15	0.0010	Significant (*p* < 0.05)

The sensitivity of the diffusion coefficient to temperature suggests that it follows the Arrhenius equation [33, 34, 35]. Consequently, the activation energy required for diffusion to occur in each capsule sample was determined.

The activation energy was found to decrease from G‐0 to G‐3 (Figure 4b) (details for activation energy calculation can be found in Supporting Information Section C). For instance, the activation energy for G‐0 was 30.69±10.25 kJ/mol, while for G‐3, it was 5.698±3.54 kJ/mol. This indicates that the porosity of the capsule significantly influences the activation energy, higher porosity results in lower activation energy. Since activation energy represents the amount of energy required to effect diffusion, a higher porosity capsule requires less energy for diffusion to occur.

Interestingly, the activation energy for the molecules of the glucose diffusion coefficient in G‐3 was higher than the ordinary Ca‐alginate, which was reported to be 14.4 kJ/mol [34]. Nevertheless, a numerical approach using nonsteady‐state dynamics was employed in our study while in the Axelsson and Persson [34] analytical methods using steady state were employed. So, the different approaches to calculating the activation energy might play a role in the two results.

### 3.3 Stability Study

The success of encapsulation is not determined by porosity alone but by stability as well. It is imperative to make sure that while the porosity is improved, the stability is also not lost.

The G‐0 sample was found to be the most resistant to the stability test, with only 8% breakage after a period of 5 h. This outcome underscores the remarkable stability of the G‐0 capsule. In the Simó et al. [36] work, the authors compared the stability of silica‐interpenetrated capsules and silica‐coated capsules employing a different methodology of stability test: compression test methodology. In this method, the strength required to reach deformation of 50% capsule is measured. But surprisingly, the study revealed that silica coating had no effect in improving the capsule stability. The authors concluded that applying the compression test method might be more suitable only for silica‐interpenetrated capsules than silica‐coated capsules. So, they recommended the method this paper reported. The improvement in stability due to silica on Ca‐alginate has been reported by many studies [37, 38, 39].

**FIGURE 5:**
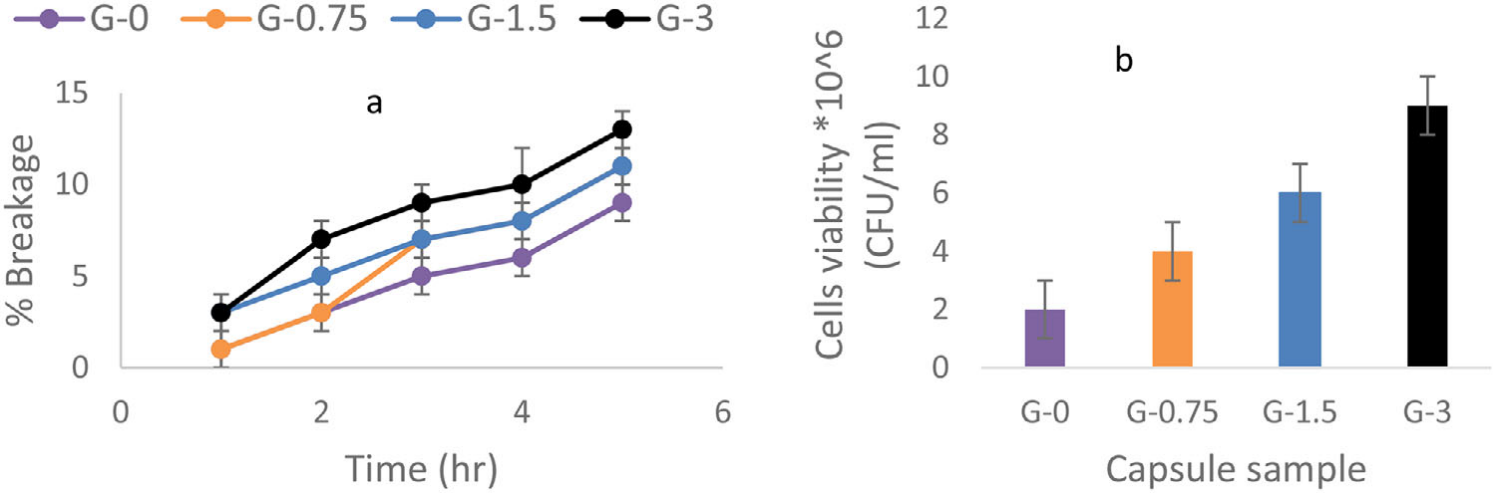
(a) Results of stability studies for conventional and porous Ca‐alginate‐silica capsules at 400 rpm and 30°C. (b) Results of viability studies of *Saccharomyces cerevisiae* encapsulated in conventional and porous Ca‐alginate‐silica capsules.

Compared to G‐0, the G‐3 sample showed the lowest resistance to the stability test, with 14% of the sample broken, as shown in Figure 5a. This observation highlights a trade‐off between enhanced porosity and stability. The traditionally silica‐coated capsule G‐0 exhibits limited diffusion properties. On the other hand, improved membrane diffusion has now resulted in reduced stability. G‐0 exhibited the greatest strength, possibly due to its compact silica coating layer. No modifications were made in the coating process of G‐0, while the other three samples G‐0.75. G‐1.5 and G‐3 were modified with PFAs 0.75, 1.5, and 3 g, respectively; this could be the potential factor that affected their stability.

To analyze the stability results statistically, a one‐way ANOVA was conducted (Table 2). After the first hour of the test, the mean difference among the capsules was found to be insignificant (*p* value 0.0762). However, for all other hour periods, the mean difference was found to be significant. Post‐hoc analysis (Figures E1–E3, Supporting Information Section E) illustrates the extent of these differences.

**TABLE 2:** Analysis of variance *p* value and mean difference of stability test.

Time (h)	*p* value	Mean difference
1	0.0698	Not significant (*p* > 0.05)
2	0.0127	Significant (*p* < 0.05)
3	0.0223	Significant (*p* < 0.05)
4	0.0000	Significant (*p* < 0.05)
5	0.0223	Significant (*p* < 0.05)

### 3.4 Microbial Encapsulation and Viability Study

To further investigate the impact of the findings of the diffusion study, *S. cerevisiae* was encapsulated in the four types of capsules. The viability of the cells (Figure 4b) inside the capsules has been found to increase with the increase in the permeability, for example, G‐3 which was found to have the highest permeability also had the highest viable cells. This suggests that the viability of the encapsulated cells is a function of capsule porosity; that is, the higher the capsule porosity, the higher the nutrients available for metabolism, hence the higher the viability of the cells. Thus, membrane porosity is a crucial factor in mass transfer resistance. In the Callone et al. [40] study, the authors reported that cell viability depends on the silica coating method employed. For instance, the *S. cerevisiae* cells were found to be constants with Ca‐alginate capsules coated by the wet method (immersing the capsules in a solution of tetraethyl orthosilicate [TEOS]) or two‐phase method (mixing the capsules with hexane to create emulsion before the TEOS application), but the cells viability increased with gas‐phase method (coating the capsules with silica gas vapor).

If the microbial cells are immobilized on the surface of the capsule, then the thin layer surrounding the biocatalyst is the only major mass transfer resistance [41]. However, if the microbes are encapsulated or entrapped within the capsule, then the internal mass transfer resistance is significant. In this system, after the nutrient concentration reaches the capsule surface, it begins the journey to the inner part of the biocatalyst. Because the diffusion is hindered by internal resistance, the concentration gradient exists within the capsule, the concentration being highest at the biocatalyst surface while it is lowest at the capsule center. But as just discussed, the membrane porosity plays a key role in terms of substrate mass transfer in microbial encapsulation. Other factors reported to influence this internal resistance are the capsule size, and the membrane thickness [39], the encapsulated cell concentration [42], cell distribution within the capsule [43], and capsule tortuosity [44].

## 4 Concluding Remarks

This study effectively demonstrated that introducing glucose as a PFA in silica‐coated alginate capsules substantially enhances their diffusion properties. For example, at 313.15 K, the diffusion coefficient of G‐0 and G‐3 was found to be $\left(3.04\pm 0.09\right)\times 10^{-3}\;\mathrm{and}\;\left(7.77\pm 0.57\right)\times 10^{-3}\;mm^{2}/\min$, respectively. A 2.6‐fold increase in the diffusion coefficient, which corresponded with a significant improvement in microbial viability. These findings offer a valuable solution to the mass transfer challenges frequently encountered in industrial fermentation processes, thereby advancing the development of more efficient and sustainable bioprocessing technologies.

Future research should consider investigating alternative PFAs or integrating this approach with other coating techniques to further optimize the balance between capsule stability and diffusion efficiency. Moreover, scaling up the production of these enhanced capsules and evaluating their performance in large‐scale bioreactors would be essential steps toward their industrial application.

## Nomenclature

*Cs*
: glucose concentration inside a capsule, g/L
*D*
: diffusion coefficient, mm^2^/min
*D*_0_
: the pre‐exponential factor, mm^2^/in
*E*_a_
: diffusion's activation energy, kJ/mol
*R*
: the gas constant, kJ/mol K
*T*
: the Kelvin temperature, K
G‐0: silica‐coated alginate capsule involving 0 g of glucose
G‐0.75: silica‐coated alginate capsule involving 0.75 g of glucose
G‐1.5: silica‐coated alginate capsule involving 1.5 g of glucose
G‐3: Silica‐coated alginate capsule involving 3 g of glucose
n
: number of samples
*r*
: capsule radius, mm
*R*′: primary alcohol
*t*
: time of diffusion, min

**Greek symbols**
Δ
: increment
∂
: partial differential
σ
: standard deviation Indices

## Author Contributions

**Bilyamin Abdulmumin:** conceptualization, methodology, writing–original draft. **Ismaila Mudi:** writing–review and editing, conceptualization, data curation. **Abdulalim Ibrahim:** supervision, review and editing, software, data curation. **Abdulwasiu Abdurrahman:** review and editing, validation, resources. **Helen Onyeaka:** review and editing, validation, data curation.

## Conflicts of Interest

The authors declare no conflicts of interest.

## Supporting information



Additional Supporting Information data associated with this article can be found in the online version at doi: 10.17632/86fc9hspxj.2.

## Data Availability

Data sharing is not applicable to this article, as no new data were created or analyzed in this study.
