# Supplementary material for: Enhancing the Diffusion Channels of Silica‐Alginate Capsules for Microbial Encapsulation
Source: Eng Life Sci. 2025 Sep 25;25(9):e70002. doi: 10.1002/elsc.70002 (PMC12461667; doi:10.1002/elsc.70002)
Supplement: Supplementary file 1 — Additional Supporting Information data associated with this article can be found in the online version at doi: 10.17632/86fc9hspxj.2. [file ELSC-25-e70002-s001.docx]

**ENHANCING THE DIFFUSION CHANNELS OF SILICA-ALGINATE CAPSULES FOR MICROBIAL ENCAPSULATION**

**Bilyamin Abdulmumin^1*^, Ismaila Mudi^2^_,_ Abdulalim Ibrahim^3,4^, Abdulwasiu Abdurrahman^5^, Helen Onyeaka^2*^**

^1^Department of Chemical Engineering, Faculty of Engineering, Ahmadu Bello University, Zaria, Nigeria,

^2^School of Chemical Engineering, University of Birmingham, Edgbaston, Birmingham B152TT, UK

^3^Chemical Engineering Department, Federal University Wukari, 670101, Nigeria.

^4^Universite de Tolouse, IMT Mines Albi, UMR CNRS 5302, Centre RAPSODEE, Campus Jalard, Albi Cedex 09 F-81013, France.

^5^Department of Chemical Engineering, Faculty of Engineering, University of Abuja, Federal Capital Territory Abuja, Nigeria

*Corresponding author email: [bilal4riid13@gmail.com](mailto:bilal4riid13@gmail.com)

**SUPPLEMENTARY MATERIALS**

**SECTION A**

**Experiment data setsandcode for fitting the experiment data, determining diffusion coefficient, and comparingexperiment with simulated as well as calculating the R-square between the experiment and simulated result.**

Table A1: Change in glucose concentration in the medium from two experiment at 30 ^o^C and 150 rpm

|  | First Experiment | | | |  | Second Experiment | | | | |
| --- | --- | --- | --- | --- | --- | --- | --- | --- | --- | --- |
| Time | G-0 | G-0.75 | G-1.5 | G-3 |  | Time | G-0 | G-0.75 | G-1.5 | G-3 |
| 0 | 1 | 1 | 1 | 1 |  | 0 | 1 | 1 | 1 | 1 |
| 15 | 0.751 | 0.669 | 0.73 | 0.7 |  | 15 | 0.855 | 0.909 | 0.77 | 0.7 |
| 30 | 0.7 | 0.662 | 0.629 | 0.559 |  | 30 | 0.802 | 0.784 | 0.771 | 0.641 |
| 45 | 0.666 | 0.65 | 0.6 | 0.45 |  | 45 | 0.766 | 0.75 | 0.7 | 0.55 |
| 60 | 0.651 | 0.52 | 0.489 | 0.425 |  | 60 | 0.651 | 0.68 | 0.611 | 0.525 |
| 75 | 0.554 | 0.489 | 0.429 | 0.434 |  | 75 | 0.616 | 0.611 | 0.531 | 0.516 |
| 90 | 0.445 | 0.411 | 0.473 | 0.393 |  | 90 | 0.545 | 0.531 | 0.473 | 0.553 |
| 105 | 0.457 | 0.471 | 0.42195 | 0.423 |  | 105 | 0.487 | 0.471 | 0.52405 | 0.523 |
| 120 | 0.392 | 0.381 | 0.463 | 0.463 |  | 120 | 0.552 | 0.561 | 0.483 | 0.483 |

Table A2: Change in glucose concentration in the capsules from two experiment at 30 ^o^C and 150 rpm

| First Experiment | | | | |  | | Second Experiment | | | | | |
| --- | --- | --- | --- | --- | --- | --- | --- | --- | --- | --- | --- | --- |
| Time | G-0 | G-0.75 | G-1.5 | G-3 |  | Time | | G-0 | G-0.75 | G-1.5 | G-3 |  |
| 0 | 0 | 0 | 0 | 0 |  | 0 | | 0 | 0 | 0 | 0 |  |
| 15 | 0.249 | 0.331 | 0.27 | 0.3 |  | 15 | | 0.145 | 0.091 | 0.23 | 0.3 |  |
| 30 | 0.3 | 0.338 | 0.371 | 0.441 |  | 30 | | 0.198 | 0.216 | 0.229 | 0.359 |  |
| 45 | 0.334 | 0.35 | 0.4 | 0.55 |  | 45 | | 0.234 | 0.25 | 0.3 | 0.45 |  |
| 60 | 0.349 | 0.48 | 0.511 | 0.575 |  | 60 | | 0.349 | 0.32 | 0.389 | 0.475 |  |
| 75 | 0.446 | 0.511 | 0.571 | 0.566 |  | 75 | | 0.384 | 0.389 | 0.469 | 0.484 |  |
| 90 | 0.555 | 0.589 | 0.527 | 0.607 |  | 90 | | 0.455 | 0.469 | 0.527 | 0.447 |  |
| 105 | 0.543 | 0.529 | 0.57805 | 0.577 |  | 105 | | 0.513 | 0.529 | 0.47595 | 0.477 |  |
| 120 | 0.608 | 0.619 | 0.537 | 0.537 |  | 120 | | 0.448 | 0.439 | 0.517 | 0.517 |  |

Table A3: Change in glucose concentration in the medium from two experiment at 35^o^C and 150 rpm

| \| First Experiment \| \| \| \| \|  \| Second Experiment \| \| \| \| \| \| --- \| --- \| --- \| --- \| --- \| --- \| --- \| --- \| --- \| --- \| --- \| \| Time \| G-0 \| G-0.75 \| G-1.5 \| G-3 \|  \| Time \| G-0 \| G-0.75 \| G-1.5 \| G-3 \| \| 0 \| 1 \| 1 \| 1 \| 1 \|  \| 0 \| 1 \| 1 \| 1 \| 1 \| \| 15 \| 0.751 \| 0.769 \| 0.71 \| 0.66 \|  \| 15 \| 0.741 \| 0.739 \| 0.71 \| 0.655 \| \| 30 \| 0.7 \| 0.662 \| 0.619 \| 0.5 \|  \| 30 \| 0.69 \| 0.662 \| 0.625 \| 0.505 \| \| 45 \| 0.646 \| 0.64 \| 0.575 \| 0.42 \|  \| 45 \| 0.646 \| 0.62 \| 0.565 \| 0.455 \| \| 60 \| 0.571 \| 0.55 \| 0.489 \| 0.415 \|  \| 60 \| 0.581 \| 0.52 \| 0.489 \| 0.425 \| \| 75 \| 0.494 \| 0.499 \| 0.429 \| 0.434 \|  \| 75 \| 0.494 \| 0.469 \| 0.429 \| 0.43 \| \| 90 \| 0.445 \| 0.411 \| 0.424 \| 0.425 \|  \| 90 \| 0.44 \| 0.411 \| 0.424 \| 0.424 \| \| 105 \| 0.457 \| 0.41 \| 0.42195 \| 0.428 \|  \| 105 \| 0.457 \| 0.411 \| 0.42195 \| 0.428 \| \| 120 \| 0.454 \| 0.407 \| 0.4269 \| 0.429 \|  \| 120 \| 0.454 \| 0.407 \| 0.4269 \| 0.428 \| |  |  |  |  |  |  |  |  |  |  |
| --- | --- | --- | --- | --- | --- | --- | --- | --- | --- | --- | --- | --- | --- | --- | --- | --- | --- | --- | --- | --- | --- | --- | --- | --- | --- | --- | --- | --- | --- | --- | --- | --- | --- | --- | --- | --- | --- | --- | --- | --- | --- | --- | --- | --- | --- | --- | --- | --- | --- | --- | --- | --- | --- | --- | --- | --- | --- | --- | --- | --- | --- | --- | --- | --- | --- | --- | --- | --- | --- | --- | --- | --- | --- | --- | --- | --- | --- | --- | --- | --- | --- | --- | --- | --- | --- | --- | --- | --- | --- | --- | --- | --- | --- | --- | --- | --- | --- | --- | --- | --- | --- | --- | --- | --- | --- | --- | --- | --- | --- | --- | --- | --- | --- | --- | --- | --- | --- | --- | --- | --- | --- | --- | --- | --- | --- | --- | --- | --- | --- | --- | --- |

Table 4A: Change in glucose concentration in the capsules from two experiment at 35^o^C and 150 rpm

|  | First experiment | | | |  | Second experiment | | | | |
| --- | --- | --- | --- | --- | --- | --- | --- | --- | --- | --- |
| Time | G-0 | G-0.75 | G-1.5 | G-3 |  | Time | G-0 | G-0.75 | G-1.5 | G-3 |
| 0 | 0 | 0 | 0 | 0 |  | 0 | 0 | 0 | 0 | 0 |
| 15 | 0.249 | 0.231 | 0.29 | 0.34 |  | 15 | 0.259 | 0.261 | 0.29 | 0.345 |
| 30 | 0.3 | 0.338 | 0.381 | 0.5 |  | 30 | 0.31 | 0.338 | 0.375 | 0.495 |
| 45 | 0.354 | 0.36 | 0.425 | 0.58 |  | 45 | 0.354 | 0.38 | 0.435 | 0.545 |
| 60 | 0.429 | 0.45 | 0.511 | 0.585 |  | 60 | 0.419 | 0.48 | 0.511 | 0.575 |
| 75 | 0.506 | 0.501 | 0.571 | 0.566 |  | 75 | 0.506 | 0.531 | 0.571 | 0.57 |
| 90 | 0.555 | 0.589 | 0.576 | 0.575 |  | 90 | 0.56 | 0.589 | 0.576 | 0.576 |
| 105 | 0.543 | 0.59 | 0.57805 | 0.572 |  | 105 | 0.543 | 0.589 | 0.57805 | 0.572 |
| 120 | 0.546 | 0.593 | 0.5731 | 0.571 |  | 120 | 0.546 | 0.593 | 0.5731 | 0.572 |

Table 5A: Change in glucose concentration in the mediumfrom two experiment at 40^o^C and 150 rpm

| First experiment | | | | |  | Second experiment | | | | |
| --- | --- | --- | --- | --- | --- | --- | --- | --- | --- | --- |
| Time | G-0 | G-0.75 | G-1.5 | G-3 |  | Time | G-0 | G-0.75 | G-1.5 | G-3 |
| 0 | 1 | 1 | 1 | 1 |  | 0 | 1 | 1 | 1 | 1 |
| 15 | 0.783 | 0.789 | 0.71 | 0.68 |  | 15 | 0.781 | 0.769 | 0.71 | 0.66 |
| 30 | 0.71 | 0.632 | 0.609 | 0.5 |  | 30 | 0.71 | 0.662 | 0.625 | 0.505 |
| 45 | 0.686 | 0.615 | 0.591 | 0.411 |  | 45 | 0.646 | 0.62 | 0.565 | 0.46 |
| 60 | 0.571 | 0.52 | 0.489 | 0.415 |  | 60 | 0.591 | 0.52 | 0.45 | 0.425 |
| 75 | 0.534 | 0.509 | 0.429 | 0.434 |  | 75 | 0.494 | 0.469 | 0.429 | 0.43 |
| 90 | 0.445 | 0.411 | 0.424 | 0.425 |  | 90 | 0.44 | 0.411 | 0.424 | 0.424 |
| 105 | 0.457 | 0.41 | 0.42195 | 0.428 |  | 105 | 0.457 | 0.411 | 0.42195 | 0.428 |
| 120 | 0.454 | 0.407 | 0.4269 | 0.429 |  | 120 | 0.454 | 0.407 | 0.4269 | 0.428 |

Table A6: Change in glucose concentration in the capsules from two experiment at 40^o^C and 150 rpm

| First experiment | | | | |  | Second experiment | | | | |
| --- | --- | --- | --- | --- | --- | --- | --- | --- | --- | --- |
| Time | G-0 | G-0.75 | G-1.5 | G-3 |  | Time | G-0 | G-0.75 | G-1.5 | G-3 |
| 0 | 0 | 0 | 0 | 0 |  | 0 | 0 | 0 | 0 | 0 |
| 15 | 0.217 | 0.211 | 0.29 | 0.32 |  | 15 | 0.219 | 0.231 | 0.29 | 0.34 |
| 30 | 0.29 | 0.368 | 0.391 | 0.5 |  | 30 | 0.29 | 0.338 | 0.375 | 0.495 |
| 45 | 0.314 | 0.385 | 0.409 | 0.589 |  | 45 | 0.354 | 0.38 | 0.435 | 0.54 |
| 60 | 0.429 | 0.48 | 0.511 | 0.585 |  | 60 | 0.409 | 0.48 | 0.55 | 0.575 |
| 75 | 0.466 | 0.491 | 0.571 | 0.566 |  | 75 | 0.506 | 0.531 | 0.571 | 0.57 |
| 90 | 0.555 | 0.589 | 0.576 | 0.575 |  | 90 | 0.56 | 0.589 | 0.576 | 0.576 |
| 105 | 0.543 | 0.59 | 0.57805 | 0.572 |  | 105 | 0.543 | 0.589 | 0.57805 | 0.572 |
| 120 | 0.546 | 0.593 | 0.5731 | 0.571 |  | 120 | 0.546 | 0.593 | 0.5731 | 0.572 |

**Matlab code to fit the experiment data, find D, predict process result, and compare the experiment and predicted results as well as calculating R-square between each experiment and predictions**

% fitDiffusion.m

% Load the experiment data

time = [0 15 30 45 60 75 90 105 120]'; % Time in minutes

G_0 = []'; % Concentration in g/L

G_0_75 = []';

G_1_5 = []';

G_3 = []';

% The radial length and number of points

R = 3; % radial length in mm

N = 10; % Number of points

dr = R / (N - 1);

% Initial concentration vector (initial concentration is zero)

C0 = zeros(N, 1);

% Initial guess for D

initialGuess = 0.01;

% Fit the data using lsqcurvefit

D_G_0 = lsqcurvefit(@(D, time) modelFunction(D, time, C0, dr, G_0(end)), initialGuess, time, G_0);

D_G_0_75 = lsqcurvefit(@(D, time) modelFunction(D, time, C0, dr, G_0_75(end)), initialGuess, time, G_0_75);

D_G_1_5 = lsqcurvefit(@(D, time) modelFunction(D, time, C0, dr, G_1_5(end)), initialGuess, time, G_1_5);

D_G_3 = lsqcurvefit(@(D, time) modelFunction(D, time, C0, dr, G_3(end)), initialGuess, time, G_3);

% Calculate R-squared values

R2_G_0 = calculateR2(time, G_0, D_G_0, C0, dr, G_0(end));

R2_G_0_75 = calculateR2(time, G_0_75, D_G_0_75, C0, dr, G_0_75(end));

R2_G_1_5 = calculateR2(time, G_1_5, D_G_1_5, C0, dr, G_1_5(end));

R2_G_3 = calculateR2(time, G_3, D_G_3, C0, dr, G_3(end));

% Display the results

fprintf('D for G-0: %.5f, R^2: %.5f\n', D_G_0, R2_G_0);

fprintf('D for G-0.75: %.5f, R^2: %.5f\n', D_G_0_75, R2_G_0_75);

fprintf('D for G-1.5: %.5f, R^2: %.5f\n', D_G_1_5, R2_G_1_5);

fprintf('D for G-3: %.5f, R^2: %.5f\n', D_G_3, R2_G_3);

% Plot the results

figure;

hold on;

plot(time, G_0, 'ro', 'DisplayName', 'G-0 Experimental');

plot(time, G_0_75, 'go', 'DisplayName', 'G-0.75 Experimental');

plot(time, G_1_5, 'bo', 'DisplayName', 'G-1.5 Experimental');

plot(time, G_3, 'ko', 'DisplayName', 'G-3 Experimental');

[~, C_model_G_0] = computeConcentration(D_G_0, time, C0, dr, G_0(end));

[~, C_model_G_0_75] = computeConcentration(D_G_0_75, time, C0, dr, G_0_75(end));

[~, C_model_G_1_5] = computeConcentration(D_G_1_5, time, C0, dr, G_1_5(end));

[~, C_model_G_3] = computeConcentration(D_G_3, time, C0, dr, G_3(end));

plot(time, C_model_G_0(:, end), 'r-', 'DisplayName', sprintf('G-0 Fit (D=%.5f, R^2=%.5f)', D_G_0, R2_G_0));

plot(time, C_model_G_0_75(:, end), 'g-', 'DisplayName', sprintf('G-0.75 Fit (D=%.5f, R^2=%.5f)', D_G_0_75, R2_G_0_75));

plot(time, C_model_G_1_5(:, end), 'b-', 'DisplayName', sprintf('G-1.5 Fit (D=%.5f, R^2=%.5f)', D_G_1_5, R2_G_1_5));

plot(time, C_model_G_3(:, end), 'k-', 'DisplayName', sprintf('G-3 Fit (D=%.5f, R^2=%.5f)', D_G_3, R2_G_3));

xlabel('Time (minutes)');

ylabel('Concentration (g/L)');

legend show;

title('Experimental Data (at 303.15 K)and Model Fits');

hold off;

% Function to integrate and compute the model concentration

function [T, C_model] = computeConcentration(D, time, C0, dr, Ceq)

[T, C_model] = ode45(@(t, C) diffusionODE(t, C, D, dr, Ceq), time, C0);

end

% Objective function for lsqcurvefit

functionC_model = modelFunction(D, time, C0, dr, Ceq)

[~, C_model] = computeConcentration(D, time, C0, dr, Ceq);

C_model = C_model(:, end); % Get the last spatial point

end

% Diffusion ODE function

functiondCdt = diffusionODE(t, C, D, dr, Ceq)

N = length(C);

dCdt = zeros(N, 1);

% Internal nodes

fori = 2:N-1

r_i = (i-1) * dr;

r_ip1 = r_i + dr/2;

r_im1 = r_i - dr/2;

dCdt(i) = D * (1 / r_i^2) * ((r_ip1^2 * (C(i+1) - C(i)) / dr) - (r_im1^2 * (C(i) - C(i-1)) / dr)) / dr;

end

% Boundary conditions

dCdt(1) = D * (C(2) - C(1)) / dr^2; % Neumann boundary condition at r=0

dCdt(N) = D * (Ceq - C(N)) / dr^2; % Fixed concentration at the outer boundary

end

% Function to calculate R^2

function R2 = calculateR2(time, C_exp, D, C0, dr, Ceq)

[~, C_model] = computeConcentration(D, time, C0, dr, Ceq);

C_model = C_model(:, end); % Get the last spatial point

SS_tot = sum((C_exp - mean(C_exp)).^2);

SS_res = sum((C_exp - C_model).^2);

R2 = 1 - (SS_res / SS_tot);

end

diffusionode.m file

functiondCdt = diffusionODE(t, C, D)

N = length(C);

dr = 1; % Assuming spatial step size is 1

dCdt = zeros(N, 1);

fori = 2:N-1

dCdt(i) = D * (C(i+1) - 2*C(i) + C(i-1)) / dr^2;

end

% Boundary conditions (assuming Neumann boundary conditions)

dCdt(1) = D * (C(2) - C(1)) / dr^2;

dCdt(N) = D * (C(N-1) - C(N)) / dr^2;

end


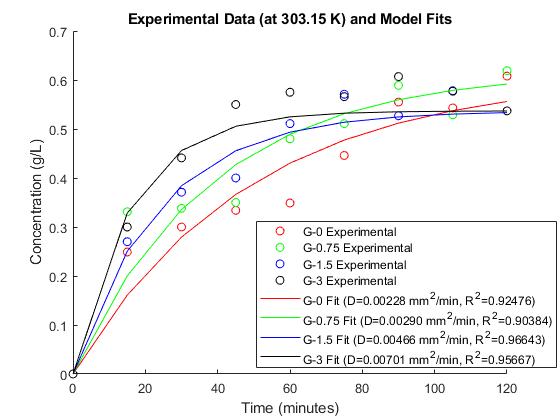


Figure A1: Comparison between the first experiment and predicted change in glucose concentration inside capsules at 30 ^o^C and 150 rpm


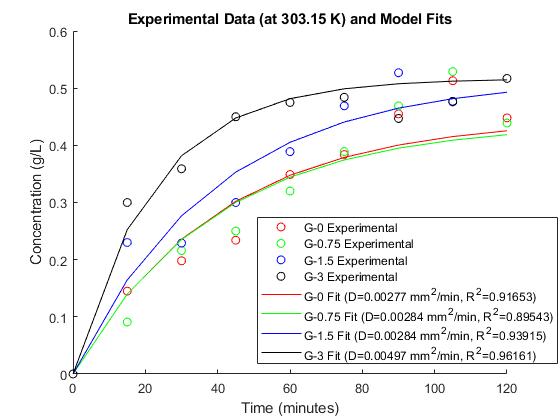


Figure A2: Comparison between the second experiment and predicted change in glucose concentration inside capsules at 30 ^o^C and 150 rpm


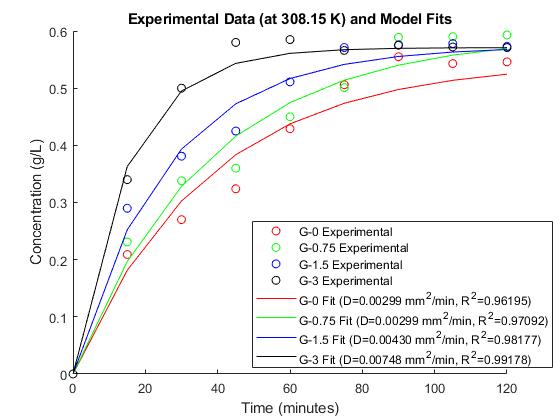


Figure A3: Comparison between the first experiment and the predicted change in glucose concentration inside capsules at 35 ^o^C and 150 rpm


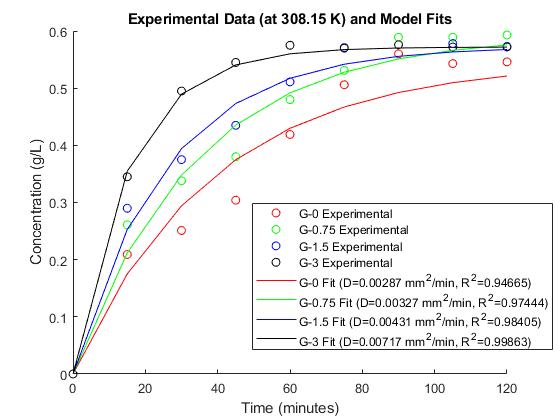


Figure A4: Comparison between the second experiment and the predicted change in glucose concentration inside capsules at 35 ^o^C and 150 rpm


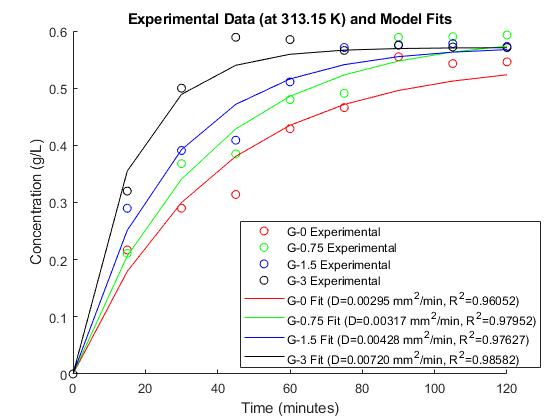


Figure A5: Comparison between the first experiment and the predicted change in glucose concentration inside capsules at 40 ^o^C and 150 rpm


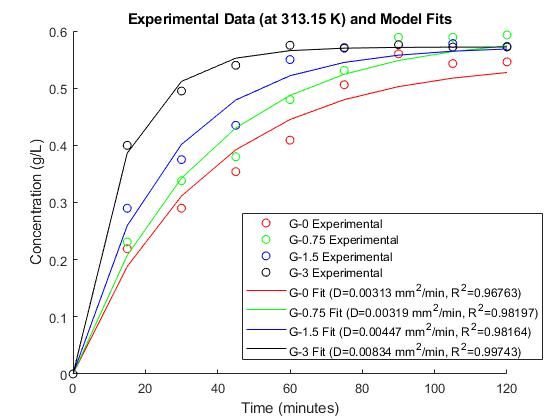


Figure A6: Comparison between the second experiment and the predicted change in glucose concentration inside capsules at 40 ^o^C and 150 rpm

Table A7: Summary of diffusion coefficients obtained from Figure A1 to 6

|  | First experiment |  |  |  |  |  |  | Second experiment |  |  |
| --- | --- | --- | --- | --- | --- | --- | --- | --- | --- | --- |
| Temperature | G-0 | G-0.75 | G-1.5 | G-3 |  |  | G-0 | G-0.75 | G-1.5 | G-3 |
| 303.15 | 0.00228 | 0.0029 | 0.00466 | 0.00701 |  |  | 0.00277 | 0.00284 | 0.00284 | 0.00497 |
| 308.15 | 0.00299 | 0.00299 | 0.0043 | 0.00748 |  |  | 0.00287 | 0.00327 | 0.00431 | 0.00717 |
| 313.15 | 0.00295 | 0.00317 | 0.00428 | 0.0072 |  |  | 0.00313 | 0.00319 | 0.00447 | 0.00834 |

Figure A7: Change in diffusion coefficient with temperature in the range of 303.15, 308.15, and 313.15 K (first experiment)

Figure A8: Change in diffusion coefficient with temperature in the range of 303.15, 308.15, and 313.15 K (Second experiment)

Table A8: Mean of the two experiment and calculated standard error (SE)

| Temperature |  | G-0 | SE | G-0.75 | SE | G-1.5 | SE | G-3 | SE |
| --- | --- | --- | --- | --- | --- | --- | --- | --- | --- |
| 303.15 |  | 0.002525 | 0.000245 | 0.00287 | 3E-05 | 0.00375 | 0.00091 | 0.00599 | 0.00102 |
| 308.15 |  | 0.00293 | 6E-05 | 0.00313 | 0.00014 | 0.004305 | 5E-06 | 0.007325 | 0.000155 |
| 313.15 |  | 0.00304 | 0.00009 | 0.00318 | 0.00001 | 0.004375 | 0.000095 | 0.00777 | 0.00057 |

Figure A9: Change in diffusion coefficient with temperature in the range of 303.15, 308.15, and 313.15 K (average experiment)

**Section B**

**Code for finding the average of the experiment and compare the average experiment with the predicted result using the average diffusion coefficient obtained in Appendix A as well as calculating the R-square between the experiment and simulated**

% Time data (same for both experiments)

time = [0 15 30 45 60 75 90 105 120]'; % Time in minutes

%First experiment at 30 ^0^C

G_0_1 = [0 0.249 0.3 0.334 0.349 0.446 0.555 0.543 0.608]'; % Concentration in g/L

G_0_75_1 = [0 0.331 0.338 0.35 0.48 0.511 0.589 0.529 0.619]';

G_1_5_1 = [0 0.27 0.371 0.4 0.511 0.571 0.527 0.57805 0.537]';

G_3_1 = [0 0.3 0.441 0.55 0.575 0.566 0.607 0.577 0.537]';

%Second experiment at 30 ^0^C

G_0_2 = [0 0.145 0.198 0.234 0.349 0.384 0.455 0.513 0.448]'; % Concentration in g/L

G_0_750_2 = [0 0.091 0.216 0.25 0.32 0.389 0.469 0.529 0.439]';

G_1_50_2 = [0 0.23 0.229 0.3 0.389 0.469 0.527 0.47595 0.517]';

G_30_2 = [0 0.3 0.359 0.45 0.475 0.484 0.447 0.477 0.517]';

%First experiment at 35 0C

G_0_1 = [0 0.249 0.3 0.354 0.429 0.506 0.555 0.543 0.546]'; % Concentration in g/L

G_0_75_1 = [0 0.231 0.338 0.36 0.45 0.501 0.589 0.59 0.593]';

G_1_5_1 = [0 0.29 0.381 0.425 0.511 0.571 0.576 0.57805 0.5731]';

G_3_1 = [0 0.34 0.5 0.58 0.585 0.566 0.575 0.572 0.571]';

%Second experiment at 35 0C

G_0_2 = [0 0.259 0.31 0.354 0.419 0.506 0.56 0.543 0.546]'; % Concentration in g/L

G_0_75_2 = [0 0.261 0.338 0.38 0.48 0.531 0.589 0.589 0.593]';

G_1_5_2 = [0 0.29 0.375 0.435 0.511 0.571 0.576 0.57805 0.5731]';

G_3_2 = [0 0.345 0.495 0.545 0.575 0.57 0.576 0.572 0.572]';

%First experiment at 40 ^0^C

G_0_1 = [0 0.217 0.29 0.314 0.429 0.466 0.555 0.543 0.546]'; % Concentration in g/L

G_0_75_1 = [0 0.211 0.368 0.385 0.48 0.491 0.589 0.59 0.593]';

G_1_5_1 = [0 0.29 0.391 0.409 0.511 0.571 0.576 0.57805 0.5731]';

G_3_1 = [0 0.32 0.5 0.589 0.585 0.566 0.575 0.572 0.571]';

%Second experiment at 40 ^0^C

Concentration in g/L

G_0_75_2 = [0 0.231 0.338 0.38 0.48 0.531 0.589 0.589 0.593]';

G_1_5_2 = [0 0.29 0.375 0.435 0.55 0.571 0.576 0.57805 0.5731]';

G_3_2 = [0 0.34 0.495 0.54 0.575 0.57 0.576 0.572 0.572]';

% First experiment at 30 or 35 or 40 0C

G_0_1 = []'; % Concentration in g/L

G_0_75_1 = []';

G_1_5_1 = []';

G_3_1 = []';

% Second experiment at 30 or 35 or 40 0C

G_0_2 = []'; % Concentration in g/L

G_0_75_2 = []';

G_1_5_2 = []';

G_3_2 = []';

% Calculate average and standard error

G_0_avg = mean([G_0_1, G_0_2], 2);

G_0_se = std([G_0_1, G_0_2], 0, 2) / sqrt(2);

G_0_75_avg = mean([G_0_75_1, G_0_75_2], 2);

G_0_75_se = std([G_0_75_1, G_0_75_2], 0, 2) / sqrt(2);

G_1_5_avg = mean([G_1_5_1, G_1_5_2], 2);

G_1_5_se = std([G_1_5_1, G_1_5_2], 0, 2) / sqrt(2);

G_3_avg = mean([G_3_1, G_3_2], 2);

G_3_se = std([G_3_1, G_3_2], 0, 2) / sqrt(2);

% Average Diffusion coefficients for each sample at 303.15 308.15 313.15 K

D_G_0 = ;% mm^2/min

D_G_0_75 = ;% mm^2/min

D_G_1_5 = ;% mm^2/min

D_G_3 = ;% mm^2/min

% Define the radial length and number of points

R = 3; % Radial length in mm

N = 10; % Number of points

dr = R / (N - 1);

% Initial concentration vector (assuming initial concentration is zero)

C0 = zeros(N, 1);

% Compute concentration for each sample using the provided D values

[~, C_model_G_0] = computeConcentration(D_G_0, time, C0, dr, G_0_avg(end));

[~, C_model_G_0_75] = computeConcentration(D_G_0_75, time, C0, dr, G_0_75_avg(end));

[~, C_model_G_1_5] = computeConcentration(D_G_1_5, time, C0, dr, G_1_5_avg(end));

[~, C_model_G_3] = computeConcentration(D_G_3, time, C0, dr, G_3_avg(end));

% Calculate R-squared values

R2_G_0 = calculateR2(time, G_0_avg, D_G_0, C0, dr, G_0_avg(end));

R2_G_0_75 = calculateR2(time, G_0_75_avg, D_G_0_75, C0, dr, G_0_75_avg(end));

R2_G_1_5 = calculateR2(time, G_1_5_avg, D_G_1_5, C0, dr, G_1_5_avg(end));

R2_G_3 = calculateR2(time, G_3_avg, D_G_3, C0, dr, G_3_avg(end));

% Plot the average data with standard error bars

figure;

hold on;

errorbar(time, G_0_avg, SE_G_0, 'ro', 'DisplayName', 'G-0 Avg ± SE');

errorbar(time, G_0_75_avg, SE_G_0_75, 'go', 'DisplayName', 'G-0.75 Avg ± SE');

errorbar(time, G_1_5_avg, SE_G_1_5, 'bo', 'DisplayName', 'G-1.5 Avg ± SE');

errorbar(time, G_3_avg, SE_G_3, 'ko', 'DisplayName', 'G-3 Avg ± SE');

% Plot the simulated data

plot(time, C_model_G_0(:, end), 'r-', 'DisplayName', sprintf('G-0 Sim (D=%.5f, R^2=%.5f)', D_G_0, R2_G_0));

plot(time, C_model_G_0_75(:, end), 'g-', 'DisplayName', sprintf('G-0.75 Sim (D=%.5f, R^2=%.5f)', D_G_0_75, R2_G_0_75));

plot(time, C_model_G_1_5(:, end), 'b-', 'DisplayName', sprintf('G-1.5 Sim (D=%.5f, R^2=%.5f)', D_G_1_5, R2_G_1_5));

plot(time, C_model_G_3(:, end), 'k-', 'DisplayName', sprintf('G-3 Sim (D=%.5f, R^2=%.5f)', D_G_3, R2_G_3));

xlabel('Time (minutes)');

ylabel('Concentration (g/L)');

legend show;

title('Average Experimental Data at 303.15 K with Simulated Results');

hold off;

% Function to integrate and compute the model concentration

function [T, C_model] = computeConcentration(D, time, C0, dr, Ceq)

[T, C_model] = ode45(@(t, C) diffusionODE(t, C, D, dr, Ceq), time, C0);

end

% Diffusion ODE function

functiondCdt = diffusionODE(t, C, D, dr, Ceq)

N = length(C);

dCdt = zeros(N, 1);

% Internal nodes

fori = 2:N-1

r_i = (i-1) * dr;

r_ip1 = r_i + dr/2;

r_im1 = r_i - dr/2;

dCdt(i) = D * (1 / r_i^2) * ((r_ip1^2 * (C(i+1) - C(i)) / dr) - (r_im1^2 * (C(i) - C(i-1)) / dr)) / dr;

end

% Boundary conditions

dCdt(1) = D * (C(2) - C(1)) / dr^2; % Neumann boundary condition at r=0

dCdt(N) = D * (Ceq - C(N)) / dr^2; % Fixed concentration at the outer boundary

end

% Function to calculate R^2

function R2 = calculateR2(time, C_exp, D, C0, dr, Ceq)

[~, C_model] = computeConcentration(D, time, C0, dr, Ceq);

C_model = C_model(:, end); % Get the last spatial point

SS_tot = sum((C_exp - mean(C_exp)).^2);

SS_res = sum((C_exp - C_model).^2);

R2 = 1 - (SS_res / SS_tot);

end


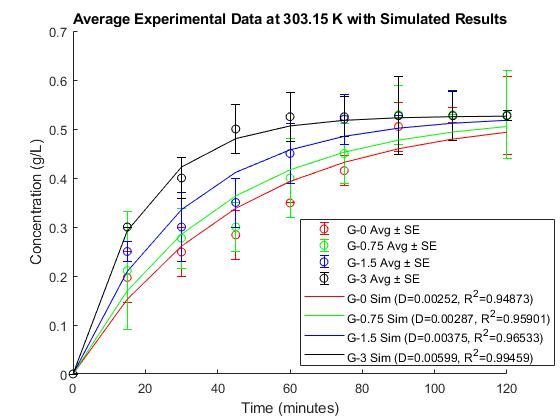


Figure B1: Change in glucose concentration inside capsules with time, comparison between experiment and predicted by Fick’s second law equation at 30 ^o^C and 150 rpm 1 g/L medium glucose concentration


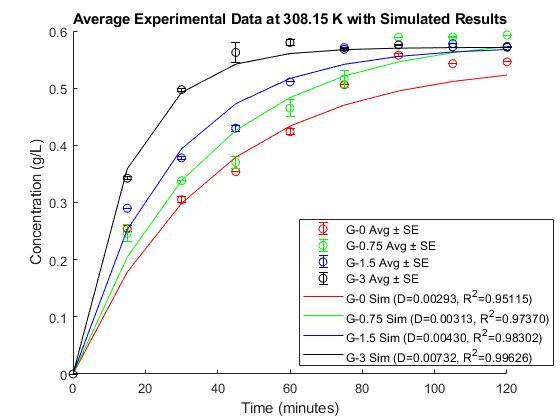


Figure B2: Change in glucose concentration inside capsules with time, comparison between experiment and predicted by Fick’s second law equation at 35 ^o^C and 150 rpm 1 g/L medium glucose concentration


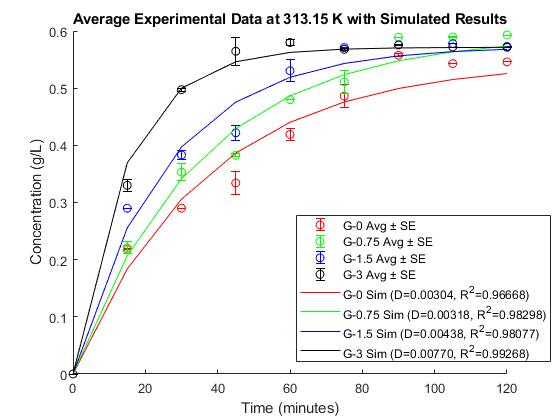


Figure B3: Change in glucose concentration inside capsules with time, comparison between experiment and predicted by Fick’s second law equation at 40 ^o^C and 150 rpm 1 g/L medium glucose concentration

**Section C**

Details analysis for calculation of activation energy from two experiments

Table C1: Summary of diffusion coefficients obtained from Figure A1 to A6

|  | First experiment | | | |  | Second experiment | | | |
| --- | --- | --- | --- | --- | --- | --- | --- | --- | --- |
| Temperature | G-0 | G-0.75 | G-1.5 | G-3 |  | G-0 | G-0.75 | G-1.5 | G-3 |
| 303.15 | 0.00228 | 0.0029 | 0.00466 | 0.00701 |  | 0.00277 | 0.00284 | 0.00284 | 0.00497 |
| 308.15 | 0.00299 | 0.00299 | 0.0043 | 0.00748 |  | 0.00287 | 0.00327 | 0.00431 | 0.00717 |
| 313.15 | 0.00295 | 0.00317 | 0.00428 | 0.0072 |  | 0.00313 | 0.00319 | 0.00447 | 0.00834 |

Figure C1: Plot the natural log of diffusion against the inverse of temperature (first experiment)

Figure C2: Plot of the natural log of diffusion against the inverse of temperature (second experiment)

Table C2: Summary of slopes from Figures C1 and C2

|  | First experiment |  |  |  |  |  | Second experiment |  |  |
| --- | --- | --- | --- | --- | --- | --- | --- | --- | --- |
| G-0 | G-0.75 | G-1.5 | G-3 |  |  | G-0 | G-0.75 | G-1.5 | G-3 |
| 2460 | 843 | 811 | 259 |  |  | 4924.7 | 4325 | 4325 | 1111.7 |
|  |  |  |  |  |  |  |  |  |  |
|  |  |  |  |  |  |  |  |  |  |
| Table C3: Summary of activation energy calculated (slope multiply by universal gas constant R (8.314 J/mol K)) |  |  |  |  |  |  |  |  |  |
|  | First experiment |  |  | Ea(kJ/mol) |  | Second experiment |  |  |  |
| G-0 | G-0.75 | G-1.5 | G-3 |  |  | G-0 | G-0.75 | G-1.5 | G-3 |
| 20.45244 | 7.008702 | 6.742654 | 2.153326 |  |  | 40.94396 | 35.95805 | 35.95805 | 9.242674 |

Table C4: Average activation energy for the two experiments and their standard error

|  |  |  |  |
| --- | --- | --- | --- |
| G-0 | G-0.75 | G-1.5 | G-3 |
| 30.6981979$\pm10.25$ | 21.48338$\pm14.47$ | 21.35035$\pm14.61$ | 5.698$\pm3.54$ |

Figure C3: Activation energy for capsules (average experiment)

**SectionD**

**Code for investigating One-Way ANOVA andsubsequent Tukey HSD using diffusion coefficients obtained from two experiments at the three temperatures**

% Data for the two experiments

data = [ ...

0.00228, 0.0029, 0.00466, 0.00701; % Experiment 1, Temp 303.15 K

0.00277, 0.00284, 0.00284, 0.00497; % Experiment 2, Temp 303.15 K

0.00299, 0.00299, 0.0043, 0.00748; % Experiment 1, Temp 308.15 K

0.00287, 0.00327, 0.00431, 0.00717; % Experiment 2, Temp 308.15 K

0.00295, 0.00317, 0.00428, 0.0072; % Experiment 1, Temp 313.15 K

0.00313, 0.00319, 0.00447, 0.00834; % Experiment 2, Temp 313.15 K

];

% Corresponding temperatures

temperatures = [303.15; 303.15; 308.15; 308.15; 313.15; 313.15];

% Group labels for capsule types

capsule_types = {'G-0', 'G-0.75', 'G-1.5', 'G-3'};

% Initialize for ANOVA and Tukey HSD

unique_temps = unique(temperatures);

% Loop through each temperature

for temp_idx = 1:length(unique_temps)

temp = unique_temps(temp_idx);

% Extract data for the current temperature

current_data = data(temperatures == temp, :);

% Reshape for ANOVA (column-wise arrangement)

reshaped_data = current_data(:);

group_labels = repelem(capsule_types, size(current_data, 1))'; % Repeat capsule labels

% Perform ANOVA

[p, tbl, stats] = anova1(reshaped_data, group_labels, 'off');

fprintf('Temperature %.2f K - ANOVA p-value: %.4f\n', temp, p);

% Perform Tukey HSD

figure;

[c, m, h, gnames] = multcompare(stats, 'CType', 'tukey-kramer');

title(sprintf('Tukey HSD for Temperature %.2f K', temp));

xlabel('Mean Difference (mm^3/min)');

ylabel('Capsule Type Comparisons');

yticks(1:length(gnames));

yticklabels(gnames);

end

Table D1: One-Way ANOVA p-values and mean difference among capsules diffusivities at different temperatures

| Temperature (K) | p-value | Mean difference |
| --- | --- | --- |
| 303.15 | 0.0762 | not significant |
| 308.15 | 0.0000 | significant |
| 313.15 | 0.0010 | significant |


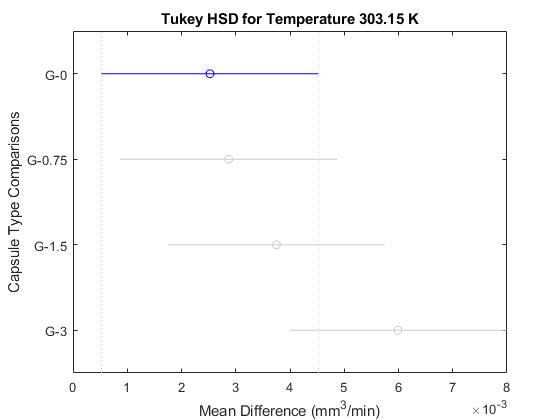


Figure D1: Multiple comparisons of mean differences of diffusion coefficients of capsules at 303.15 K


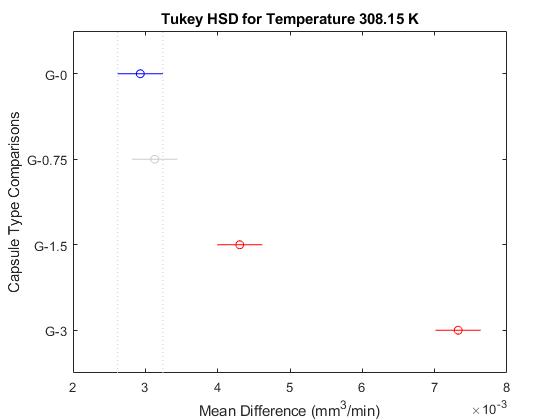


Figure D2: Multiple comparisons of mean differences of diffusion coefficients of capsules at 308.15 K


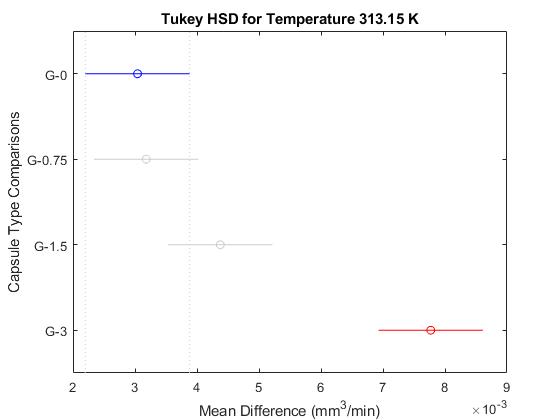


Figure D3: Multiple comparisons of mean differences of diffusion coefficients of capsules at 313.15 K

**SectionE**

**Code for investigating ONE-Way Anova andsubsequent Tukey HSD for capsules stability test given in Table E1**

% Define the percentage breakage data for two sets of experiments

data = [ ...

2, 2, 4, 4; % Time 1 (Set 1)

4, 4, 6, 8; % Time 2 (Set 1)

6, 8, 8, 10; % Time 3 (Set 1)

6, 8, 8, 10; % Time 4 (Set 1)

10, 12, 12, 14; % Time 5 (Set 1)

1, 1, 3, 3; % Time 1 (Set 2)

3, 3, 5, 7; % Time 2 (Set 2)

5, 7, 7, 9; % Time 3 (Set 2)

6, 8, 8, 10; % Time 4 (Set 2)

9, 11, 11, 13; % Time 5 (Set 2)

];

% Corresponding time points

time_points = [1; 2; 3; 4; 5; 1; 2; 3; 4; 5];

% Group labels for capsule types

capsule_types = {'G-3', 'G-1.5', 'G-0.75', 'G-0'};

% Initialize for ANOVA

unique_times = unique(time_points);

% Loop through each time point

fortime_idx = 1:length(unique_times)

time = unique_times(time_idx);

% Extract data for the current time point

current_data = data(time_points == time, :);

% Reshape for ANOVA (column-wise arrangement)

reshaped_data = current_data(:);

group_labels = repelem(capsule_types, size(current_data, 1))'; % Repeat capsule labels

% Perform ANOVA

[p, tbl, stats] = anova1(reshaped_data, group_labels, 'off');

fprintf('Time %.1f hr - ANOVA p-value: %.4f\n', time, p);

% Perform Tukey HSD

figure;

[c, m, h, gnames] = multcompare(stats, 'CType', 'tukey-kramer');

title(sprintf('Tukey HSD for Time %.1f hr', time));

xlabel('Mean Difference (Percentage Breakage)');

ylabel('Capsule Type Comparisons');

yticks(1:length(gnames));

yticklabels(gnames);

end

**Table E1: Percentage capsule breakage for two experiments at 400 rpm and 30^0^C over five hours time period**

| Time (hr) | G-0 | G-075 | G-1.5 | G-3 |  | G-0 | G-075 | G-1.5 | G-3 |
| --- | --- | --- | --- | --- | --- | --- | --- | --- | --- |
| 1 | 2 | 2 | 4 | 4 |  | 1 | 1 | 3 | 3 |
| 2 | 4 | 4 | 6 | 8 |  | 3 | 3 | 5 | 7 |
| 3 | 6 | 8 | 8 | 10 |  | 5 | 7 | 7 | 9 |
| 4 | 6 | 8 | 8 | 10 |  | 6 | 8 | 8 | 10 |
| 5 | 10 | 12 | 12 | 14 |  | 9 | 11 | 11 | 13 |

Table E2: One-way ANOVA results for p-values and mean difference at each time

| Time (hr) | p-value | Mean difference |
| --- | --- | --- |
| 1 | 0.0698 | Not significant |
| 2 | 0.0127 | Significant |
| 3 | 0.0223 | Significant |
| 4 | 0.0000 | Significant |
| 5 | 0.0223 | Significant |


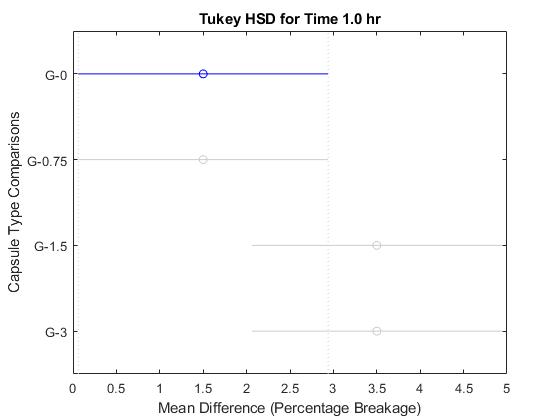


Figure E1: Multiple comparisons of means difference at the first hour


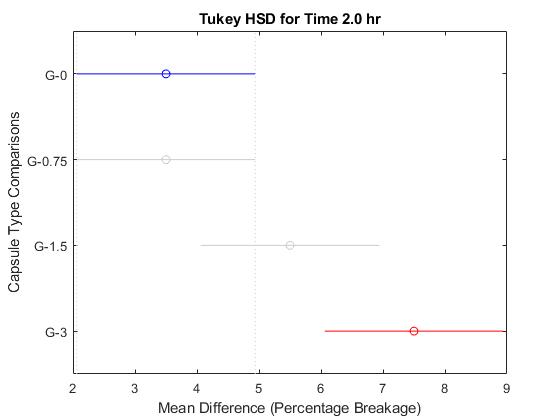


Figure E2: Multiple comparisons of means difference at the second hour


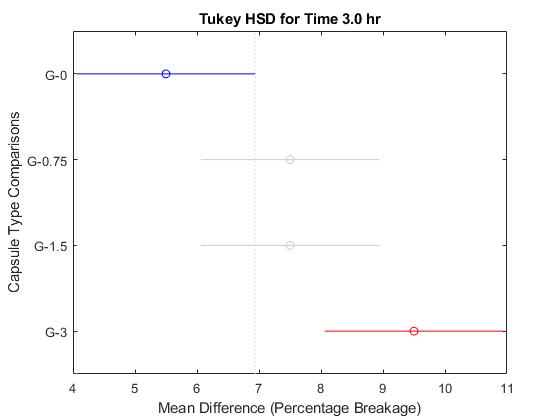


Figure E3: Multiple comparisons of means difference at the third hour of the test


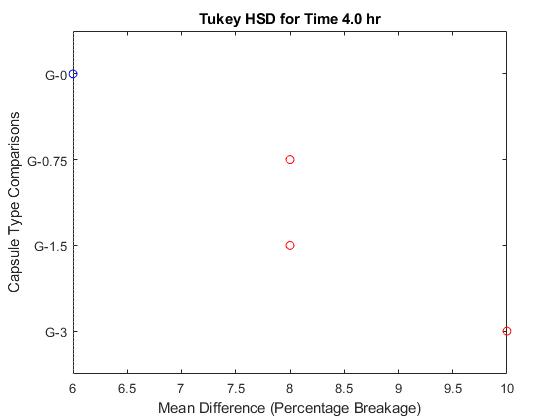


Figure E4: Multiple comparisons of means difference at the fourth hour


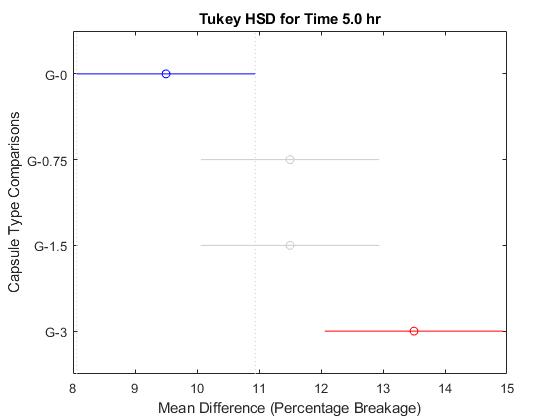


Figure E5: Multiple comparisons of means difference at the fifth hour

**SectionF**

**Microscopes images of capsules G-0, G-0.75, G-1.5 and G-3**


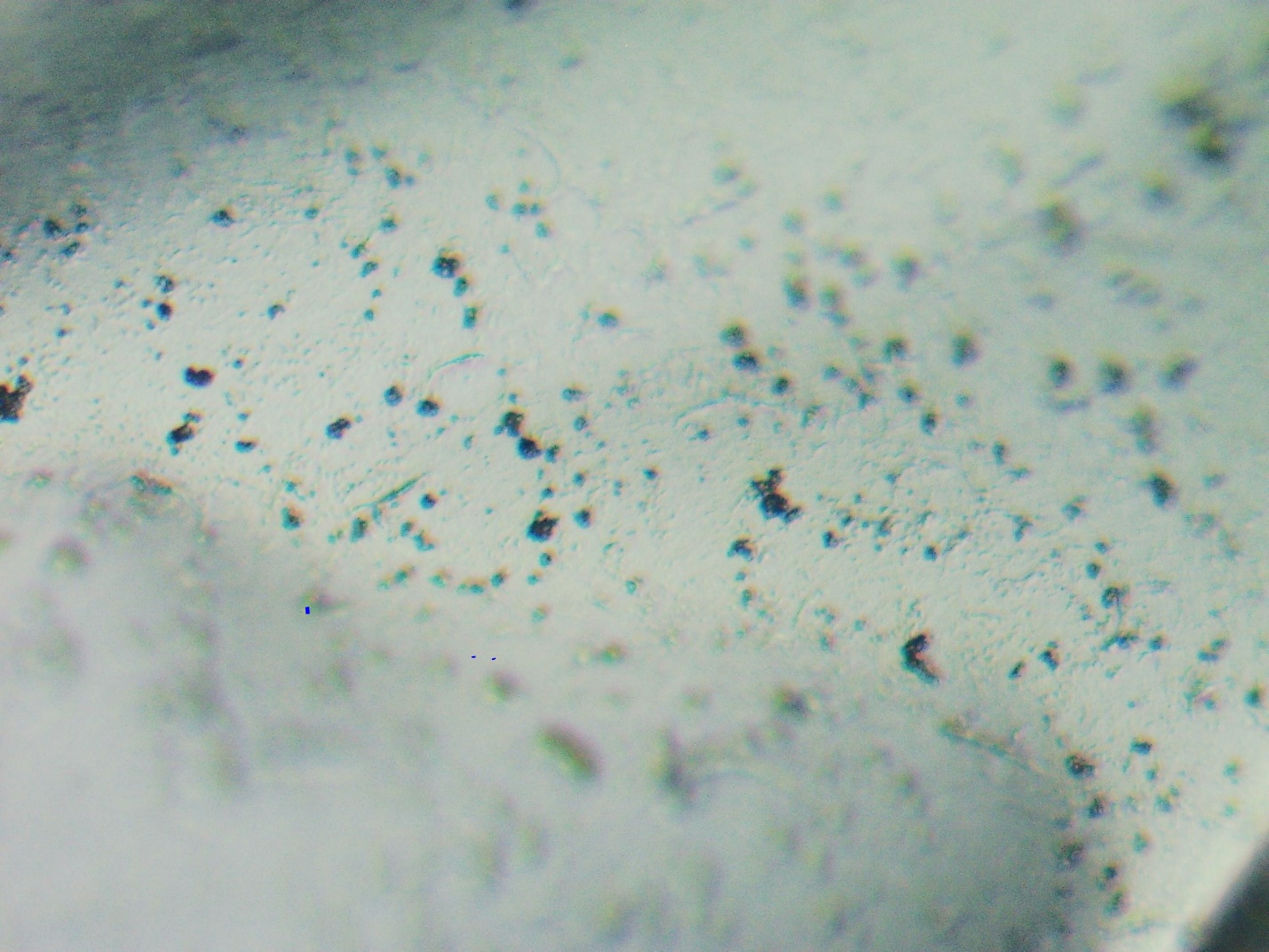


G-0


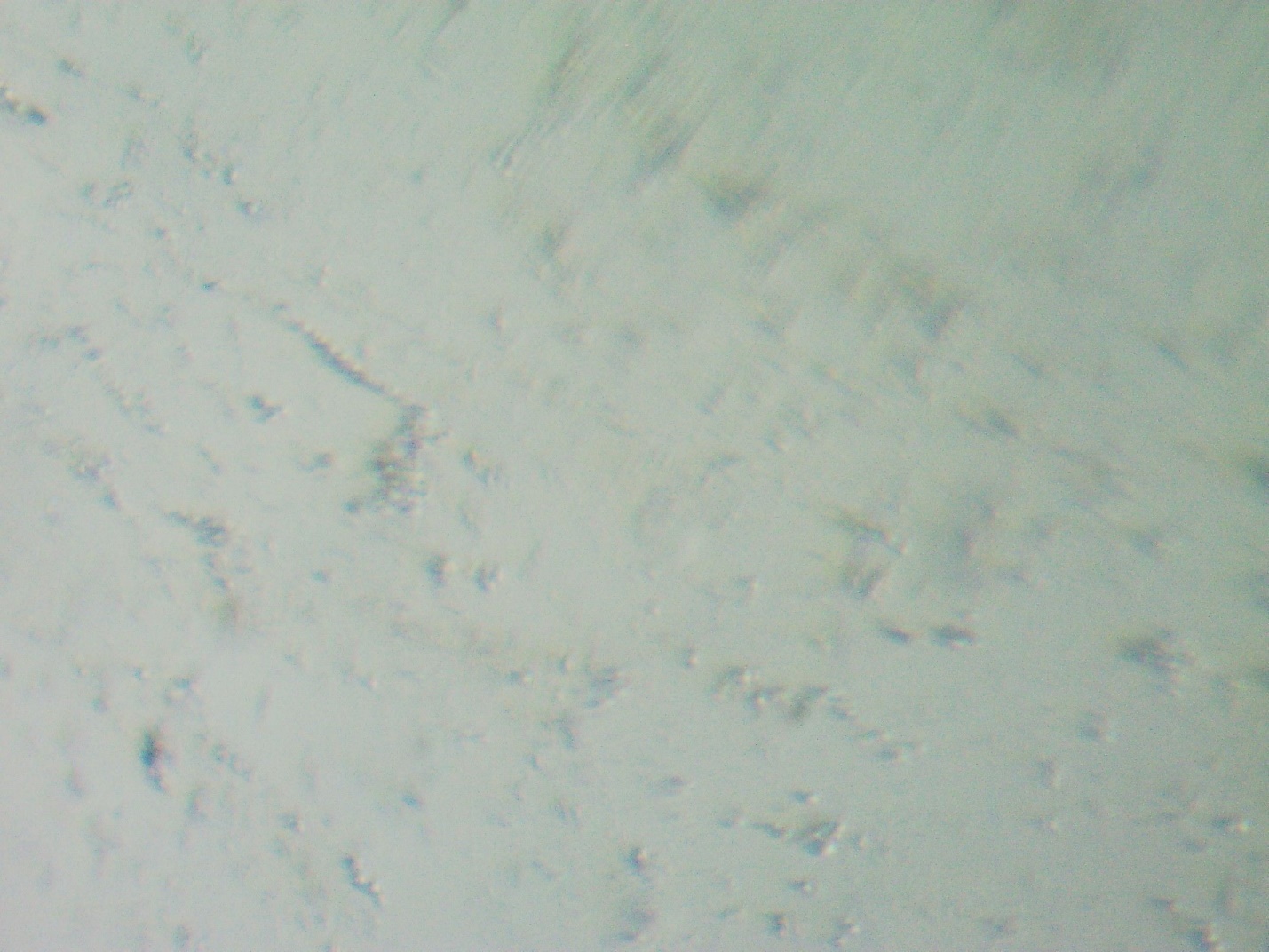


G-0.75


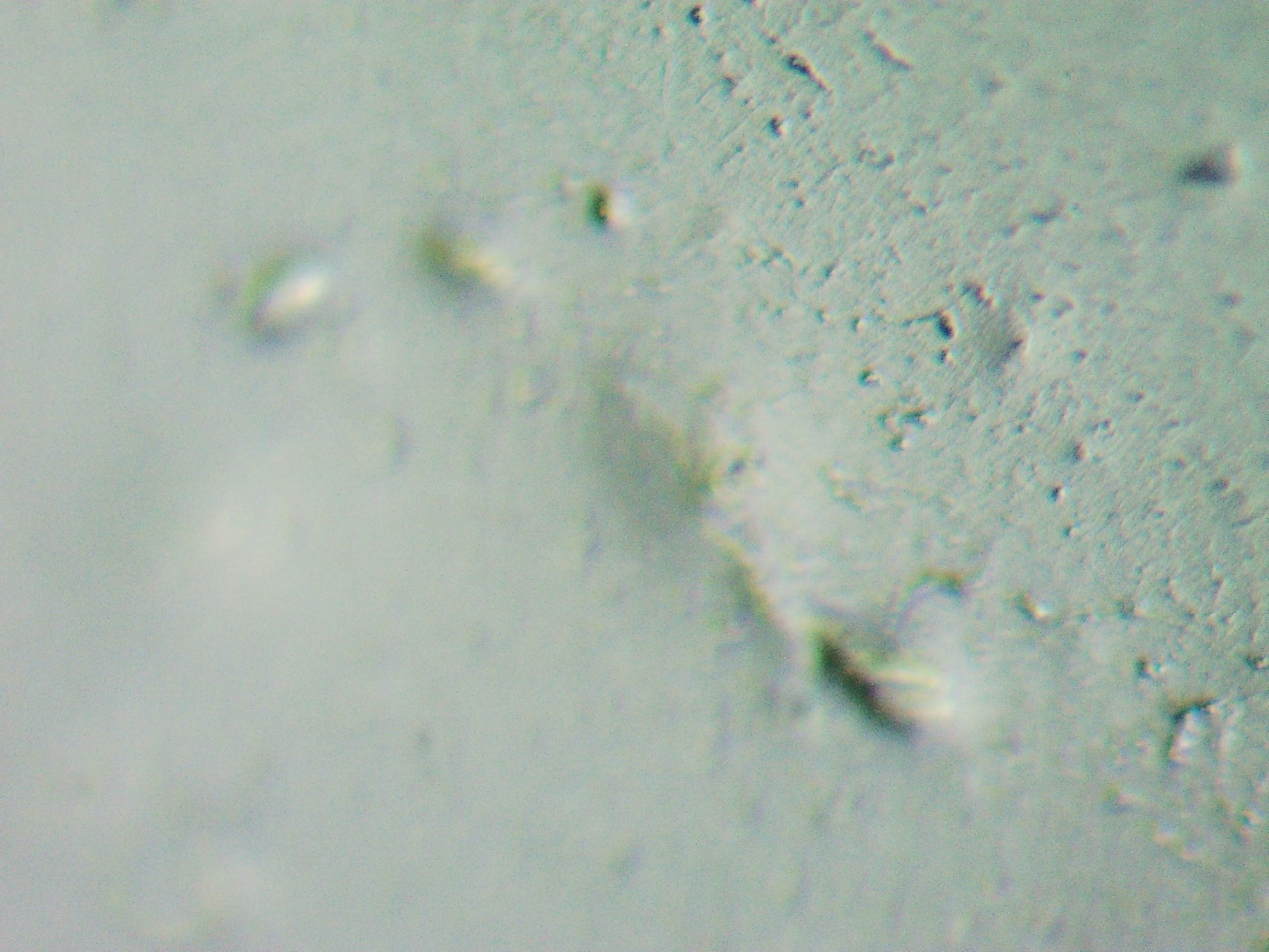


G-0.75


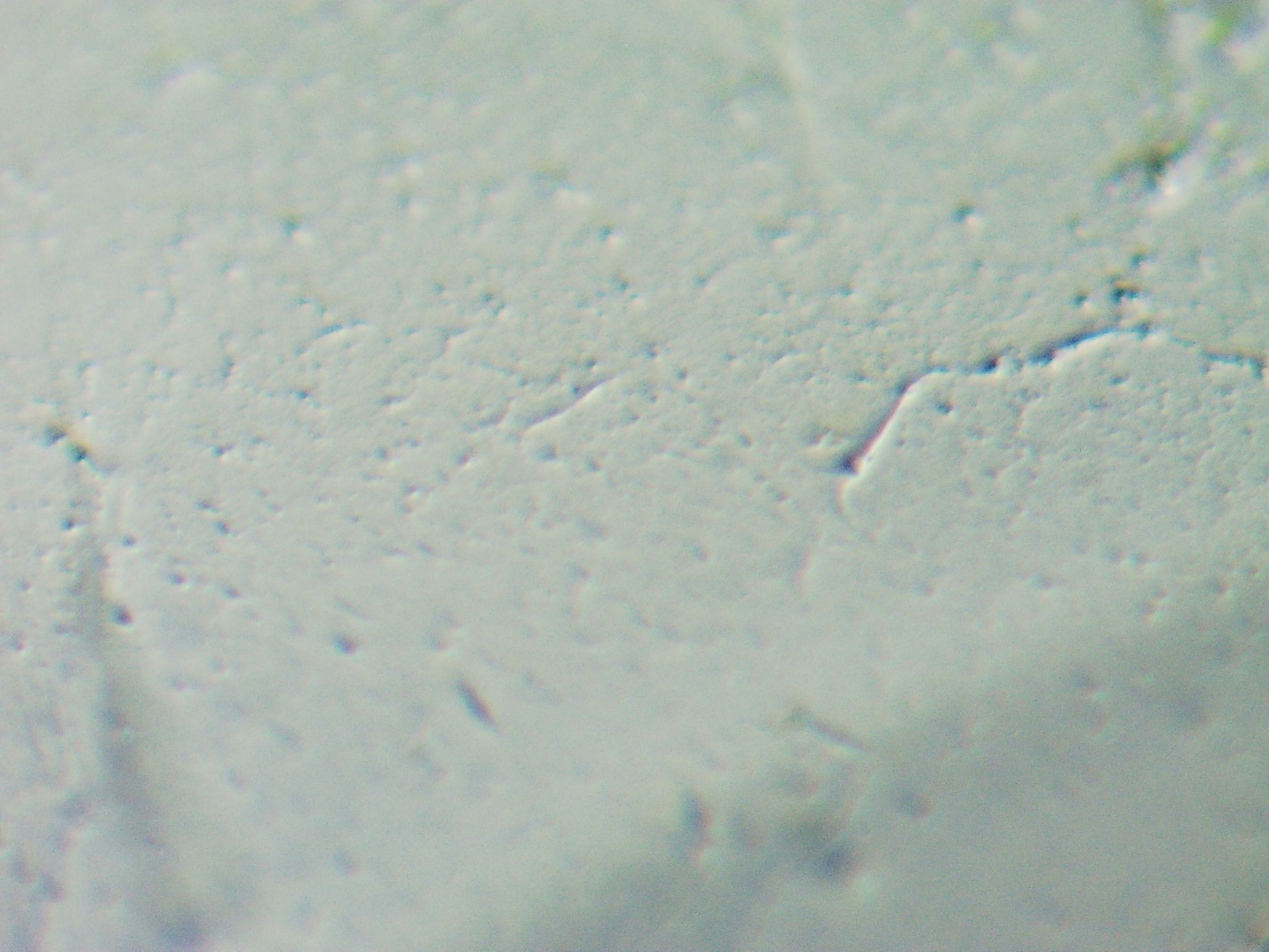


G-3
